# TDP43 promotes stemness of breast cancer stem cells through CD44 variant splicing isoforms

**DOI:** 10.1038/s41419-022-04867-w

**Published:** 2022-05-03

**Authors:** Lu Guo, Hao Ke, Honglei Zhang, Li Zou, Qin Yang, Xuemei Lu, Limin Zhao, Baowei Jiao

**Affiliations:** 1 State Key Laboratory of Genetic Resources and Evolution, Kunming Institute of Zoology, Chinese Academy of Sciences, Kunming, 650201 Yunnan China; 2 Kunming College of Life Science, University of Chinese Academy of Sciences, Kunming, 650201 China; 3 Human Aging Research Institute (HARI) and School of Life Science, Nanchang University, and Jiangxi Key Laboratory of Human Aging, Nanchang, 330031 Jiangxi China; 4 Center for Scientific Research, Yunnan University of Chinese Medicine, Kunming, 650500 Yunnan China; 5 KIZ-CUHK Joint Laboratory of Bioresources and Molecular Research in Common Diseases, Kunming Institute of Zoology, Chinese Academy of Sciences, Kunming, 650223 Yunnan China

**Keywords:** Cancer stem cells, Breast cancer

## Abstract

Alternative splicing (AS) is a promising clinical target for cancer treatment at the post-transcriptional level. We previously identified a unique AS profile in triple-negative breast cancer (TNBC), which is regulated by the splicing regulator TAR DNA-binding protein-43 (TDP43), thus indicating the crucial role of TDP43 in heterogeneous TNBC. Cluster of differentiation 44 (CD44), a widely recognized marker for breast cancer stem cells (BCSCs), is extensively spliced into CD44 variant AS isoforms (CD44v) during the development of breast cancer. At present, however, the regulatory mechanism of CD44v is not fully understood. In the current study, we found that loss of TDP43 inhibits BCSC stemness by reducing the abundance of CD44v. In addition, serine-arginine-rich splicing factor 3 (SRSF3), another splicing factor and partner of TDP43, acts as an upstream regulator of TDP43 to maintain CD44v isoforms and thereafter BCSC stemness. Mechanistically, SRSF3 stabilizes the mRNA of TDP43 by inhibiting nonsense-mediated decay (NMD). These findings illustrate the important role of complicated regulatory networks formed by splicing factors in TNBC progression, thus providing potential therapeutic targets from an AS perspective.

## Introduction

Breast cancer has surpassed lung cancer as the most diagnosed cancer and ranks fourth among cancer-related deaths [1]. As a small subpopulation, breast cancer stem cells (BCSCs), which exhibit self-renewal and differentiation capacity, contribute to tumor heterogeneity, recurrence, and progression [2]. The transmembrane protein CD44 is widely recognized as a BCSC marker in breast and other cancers [3, 4]. As CD44 is overexpressed (OE) in BCSCs and responsible for various aspects of cancer progression, such as CSC stemness, tumor recurrence, and metastasis [5], it is also considered a promising target for cancer treatment [6]. CD44v6-targeted therapy has been approved for clinical trials [6]. CD44 is encoded by 19 exons on chromosomal 13 in human, and 20 exons in mice. It is alternatively spliced resulting in the CD44s (standard) isoform and the CD44v (variant) isoforms. CD44s consists of exons 1–5 and 16–20 (standard exons), while CD44v consists of standard exons and alternative exons 6–15 (variable exons). These variants are numbered v1 to v10, out of which v1 isoform is not encoded in human [7]. CD44v contain extended extracellular domain that can interact with growth factor and cytokines in microenvironment [8]. Breast cancer subpopulations overexpressing CD44v are reported to have more aggressive phenotypes [4, 9]. For example, the CD44v3, v5, and v6 isoforms are OE in human breast tumor samples, but not in normal mammary ducal and hyperplastic lesions, and are positively correlated with breast cancer lung metastasis and poor prognosis [10, 11]. The switch from CD44s to CD44v increases the stemness of BCSCs [12] and the ectopic expression of CD44s inhibits breast cancer stemness [13], highlighting the crucial roles of CD44v in breast BCSCs. Moreover, CD44v containing exons 8 to 10 (CD44v8-10), the most abundant variable exons in human carcinomas, including breast cancer [14, 15], are highly associated with proliferation, metastasis, stemness, poor prognosis, and reactive oxygen species resistance in cancer cells [16–21]. Therefore, the CD44v isoforms, especially CD44v8-10, are promising therapeutic targets for breast cancer treatment. Further research in deciphering CD44 AS regulation is necessary to provide a theoretical basis for clinical application. However, although several splicing factors modulating CD44v have been identified, the mechanism underlying CD44 AS, especially CD44v8-10 AS, in breast cancer remains unclear. Thus, understanding the regulation of CD44 isoform generation should help clarify the underlying mechanism for clinical application.

AS is a widespread and essential cellular process regulated by cis-elements located on pre-mRNA and trans-splicing factors [22]. Although the generation of splicing code based on cis-elements has been explored [23], transfactors have also been widely investigated as new participants of splicing in terms of their mutation and gene expression levels [24–26], leading to functional and non-functional end-products by aberrant AS. Splicing factors are significantly associated with tumorigenesis, development, and treatment failure [27]. While aberrant splicing at pre-mRNA cis-elements is susceptible to drug resistance, splicing factors control a series of AS events and may be much more robust to drug resistance [28], indicating the considerable clinical potential of splicing factors. To date, many splicing factors have been investigated in clinical trials [29, 30]. The splicing factor SRSF3, which is the smallest member of the serine/arginine rich protein family, is a proto-oncogene OE in many cancers, including breast cancer, and is known to regulate cancer progression, metastasis, prognosis, and drug resistance [31, 32]. SRSF3 promotes exon skipping of HER2 exon 20 to generate the Δ16HER2 isoform, which increases proliferation, invasion, and resistance to trastuzumab treatment [26]. The splicing factor TDP43, which is highly expressed in various tumors, regulates many AS events and is a vital factor in breast cancer progression [32–34]. Several compounds affect the expression levels of SRSF3 and TDP43, and thus both molecules could act as potential therapeutic targets for tumors [35, 36].

In comparison to other subtypes, triple-negative breast cancer (TNBC) shows high malignancy and poor prognosis due to its high heterogeneity and enrichment of BCSCs. We previously identified a unique homogenous AS profile in TNBC [32], with TDP43 found to be responsible for this profile via regulation of *NUMB* and *PAR3* AS. In this study, TDP43 was identified as an important factor participating in the maintenance of BCSC stemness by increasing CD44v inclusion, especially CD44v8-10. In addition, SRSF3 was found to increase TDP43 abundance and thereafter modulate CD44 AS and BCSC stemness. Our work highlights the regulatory mechanism of CD44 as a network of splicing factors.

## Results

### Knockdown (KD) of TDP43 inhibits BCSC stemness

**Fig. 1:**
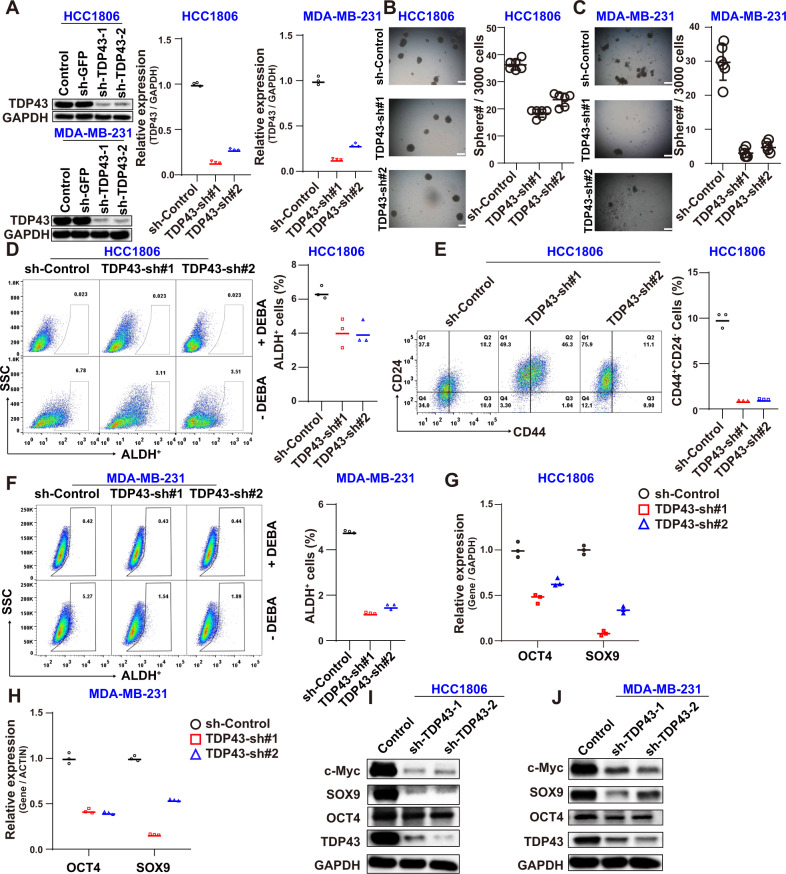
Loss of TDP43 inhibits stemness of BCSCs. **A** Western blot (left) and qPCR (right) showing the knockdown (KD) efficiency in MDA-MB-231 and HCC1806 cells after TDP43 KD. **B**, **C** Calculation of number of tumorspheres in MDA-MB-231 and HCC1806 cells upon TDP43 KD. ALDH cell fraction (**D**) and CD24^low^CD44^high^ population (**E**) in HCC1806 cells, and ALDH ratios in MDA-MB-231 cells (**F**) by flow cytometry analysis. **G**, **H** Expression changes in stemness markers. **I**, **J** KD efficiency and stemness genes at protein level. Data are means ± SD.

We previously ascertained the crucial role of TDP43 in breast cancer progression [32]. Here, to further explore the role of TDP43 in BCSCs, we examined cancer stemness upon KD of TDP43 in TNBC cell lines MDA-MB-231 and HCC1806. The short-hairpin RNAs (shRNAs) targeting TDP43 demonstrated high KD efficiency (Fig. 1A and WB). Our assays detecting BCSC stemness indicated that TDP43 KD significantly reduced the number of tumorspheres in both the HCC1806 and MDA-MB-231 cells (Fig. 1B, C). With well-recognized markers for BCSCs [37, 38], our cell fraction ratio assay using flow cytometry showed that the ratios of the ALDH+ and CD24^low^CD44^high^ cell fractions decreased markedly upon TDP43 KD in HCC1806 cells (Fig. 1D, E). As MDA-MB-231 cells are almost all positive for CD44 staining, we checked the ALDH+ populations in the MDA-MB-231 cells and found consistent results with those obtained from the HCC1806 cell line (Fig. 1F). Moreover, we examined the expression level of stemness-associated genes after TDP43 KD. Results showed that the mRNA (Fig. 1G, H) and protein expression levels (Fig. 1I, J and WB) of stem cell markers were significantly down-regulated in the MDA-MB-231 and HCC1806 cells. These data suggest that TDP43 plays an important role in the maintenance of BCSC stemness.

### TDP43 regulates CD44 AS

**Fig. 2:**
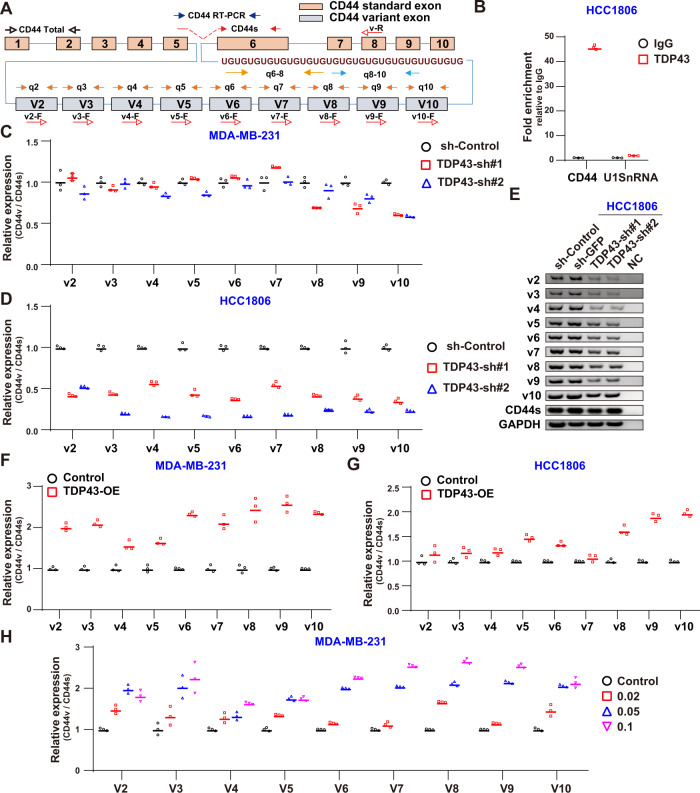
TDP43 regulates AS of CD44. **A** Schematic of CD44 pre-mRNA structure and TDP43-binding motif. Arrows indicate primers for qPCR (q) and RT-PCR (vx-F and v-R). **B** Binding of CD44 mRNA to TDP43 proteins detected by RIPA in HCC1806 cells. **C**, **D** qPCR analysis of changes in CD44v/s following TDP43 KD. **E** Semi-quantitative RT-PCR of CD44 isoforms upon TDP43 KD. The primer pairs (vx-F and v-R) are applied for CD44v amplification. e.g., v2 band is amplified using v2-F and v-R primers. **F**, **G** qPCR analysis of CD44v/s ratio following TDP43 OE. **H** qPCR analysis of CD44v/s changes at various TDP43 concentrations by doxycycline induction (μg/ml). Data are means ± SD.

In our earlier study, we demonstrated that TDP43 regulates global AS and is responsible for the unique splicing profile in TNBC [32]. Therefore, we speculated that TDP43 may regulate BCSCs through AS. As a commonly used BCSC marker, CD44 undergoes extensive splicing to generate hundreds of different molecules. Here, the CD44 pre-mRNA sequences were analyzed and the (UG)_18_ repeat sequence was found, which is a TDP43-binding motif between variant exon 10 and standard exon 6 (Fig. 2A) [39]. To verify this binding potential, we performed RNA immunoprecipitation qPCR assays (RIP-qPCR) and found remarkable enrichment of *CD44* mRNA interactions with TDP43 proteins (Fig. 2B). To determine whether TDP43 regulates *CD44* AS, primers for quantitative real-time polymerase chain reaction (qPCR), reverse-transcription PCR (RT-PCR), and semi-quantitative RT-PCR were designed to detect individual CD44 exons (Fig. 2A), and the CD44v to CD44s ratio was calculated to assess relative fluctuation in CD44v abundance [10]. After KD of TDP43, the transcription levels of CD44 (total) were unchanged (Fig. S1A), but the levels of v8, v9, and v10 decreased compared with the levels of CD44s in the MDA-MB-231 cells (Fig. 2C). The CD44v to CD44s ratio was also examined in the HCC1806 cells, which showed that CD44v isoform abundance significantly declined after TDP43 KD (Fig. 2D). The AS CD44v changes were also validated by semi-quantitative RT-PCR (Fig. 2E), further confirming the downregulation of CD44v abundance. The decrease in CD44 (total) in the HCC1806 cells (Fig. S1B) indicates that CD44 transcription may be regulated by TDP43 acting as a transcription factor [40]. The expression levels of CD44v6-8 and CD44v8-10 were also detected after KD of TDP43 and found to be significantly suppressed in both cell lines (Fig. S1C, D). These results indicate that TDP43 regulates BCSC stemness through CD44 AS. To clarify the above regulatory roles, TDP43 was OE in the HCC1806 and MDA-MB-231 cells (Fig. S1E). While total CD44 levels were generally stable in both cell lines (Fig. S1F), all variant exons were markedly increased in the MDA-MB-231 cells and variant exons 8 to 10 were significantly up-regulated in the HCC1806 cells after TDP43 OE (Fig. 2F, G). We next increased the TDP43 expression levels in a dose-dependent manner using a doxycycline-induction system. Results showed that TDP43 levels were greatly induced by different concentrations of doxycycline (Fig. S1G), and CD44v abundance also increased substantially in a dose-dependent manner (Fig. 2H), further confirming the regulatory roles of TDP43 in CD44 AS. Overall, the above assays demonstrate that TDP43 promotes CD44 variants, especially CD44v8, v9, and 10 exon inclusion.

### SRSF3 maintains BCSC stemness

**Fig. 3:**
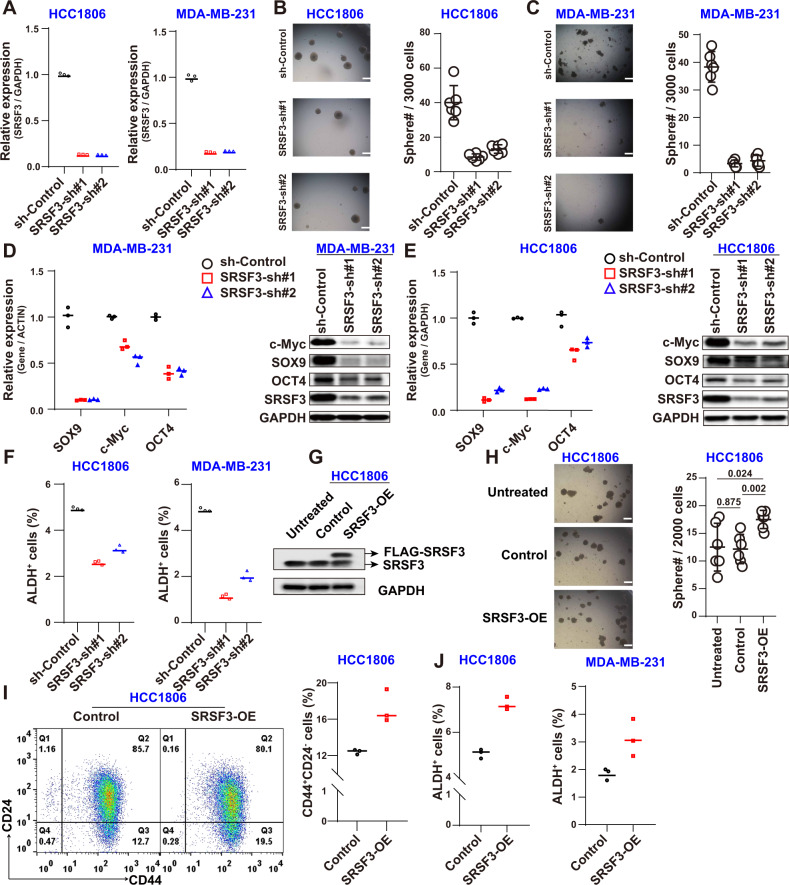
SRSF3 maintains stemness of BCSCs. **A** qPCR analysis of SRSF3 KD efficiency in HCC1806 and MDA-MB-231 cells. **B**, **C** Comparison of number of tumorspheres in MDA-MB-231 and HCC1806 cells upon SRSF3 KD. **D**, **E** Examination of stemness marker (SOX9, c-Myc, and OCT4) at RNA and protein levels upon SRSF3 KD in MDA-MB-231 and HCC1806 cells. **F** ALDH analysis upon SRSF3 KD in HCC1806 and MDA-MB-231 cells. **G** Western blotting analysis of SRSF3 protein levels in HCC1806 cells. **H** Tumorsphere assay upon SRSF3 OE. The unpaired *t*-test was used to compare the number of sphere among groups. **I** Flow cytometry analysis of ratios of CD44+/CD24−/low population after SRSF3 OE. **J** ALDH analysis following SRSF3 OE in HCC1806 and MDA-MB-231 cells. Data are means ± SD.

Next, to explore whether SRSF3 regulate stemness of BCSCs, we detected cancer stemness after SRSF3 interference by shRNAs in both the MDA-MB-231 and HCC1806 cell lines. The SRSF3 KD efficiencies are shown in Fig. 3A. Results indicated that the number of tumorspheres were significantly reduced following KD of SRSF3 (Fig. 3B, C). To further explore the underlying mechanism of SRSF3 in regulating BCSC stemness, we detected various stemness-related markers and found that SOX9, c-Myc, and OCT4 were significantly down-regulated in the MDA-MB-231 and HCC1806 cells (Fig. 3D, E and WB). The BCSC markers were also detected using flow cytometry, which showed that the ratios of the ALDH+ cell fractions clearly decreased upon SRSF3 KD (Figs. 3F and S2A). To clarify its role in BCSCs, SRSF3 was also OE in the MDA-MB-231 and HCC1806 cells (Fig. 3G and WB). The tumorsphere assay indicated that BCSC stemness increased significantly upon SRSF3 OE (Fig. 3H). Stemness was also examined using flow cytometry. Results showed that the cell fractions of CD24^low^CD44^high^ in the HCC1806 cells (Fig. 3I) and ALDH+ in the MDA-MB-231 and HCC1806 cells increased (Figs. 3J and S2B). Similar results were also observed in MCF7 cells (Figs. S2C–E and WB). Thus, these results suggest that SRSF3 is an important factor for the maintenance of BCSC stemness.

### SRSF3 regulates CD44 AS in breast cancer cells

**Fig. 4:**
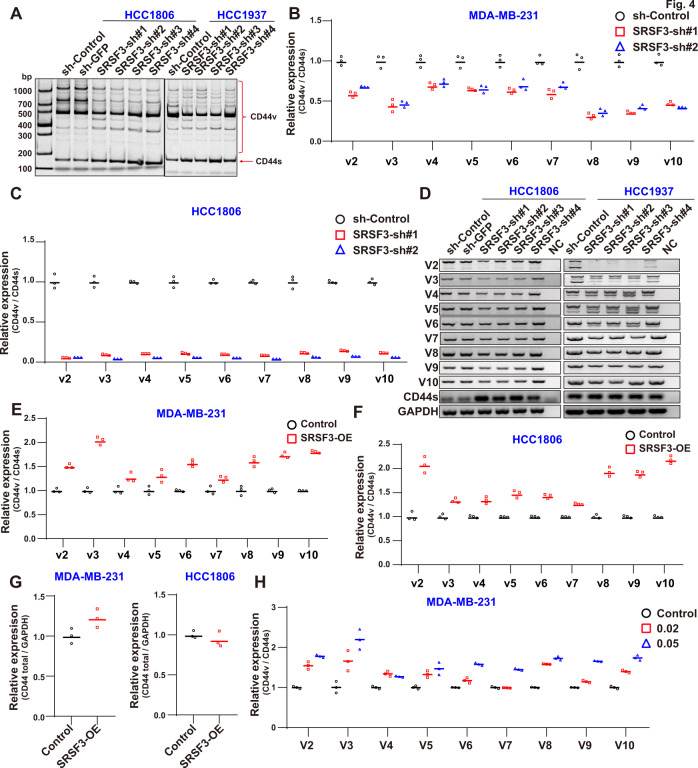
SRSF3 regulates CD44 AS in breast cancer cells. **A** Semi-quantitative RT-PCR (CD44 RT-PCR) of CD44 isoforms after SRSF3 KD in HCC1806 and HCC1937 cells. **B**, **C** CD44v/s analysis by qPCR after SRSF3 KD. **D** CD44 splicing events verified by semi-quantitative RT-PCR (primers vx-F and v-R). **E**, **F** CD44v/s analysis by qPCR upon SRSF3 OE. **G** mRNA expression of CD44 (total) in MDA-MB-231 and HCC1806 cells upon SRSF3 OE. **H** qPCR analysis of changes in CD44v/s under various SRSF3 concentrations in MDA-MB-231 cells. Data are means ± SD.

We next investigated whether SRSF3 regulates BCSC stemness via AS. Due to the inconsistency of previous studies on CD44 AS by SRSF3 [41, 42], we depleted SRSF3 in cells with different levels of CD44v expression using four shRNA pairs with different KD efficiencies (Fig. S3A, B and WB). Results indicated that loss of SRSF3 reduced the CD44v isoforms but increased the CD44s isoform in the HCC1806, HCC1937 (Fig. 4A), and MDA-MB-468 cells (Fig. S3C), suggesting that SRSF3 KD induces a switch from CD44v to CD44s.

To further clarify the influence of SRSF3 on CD44 AS, we determined the expression of each variant exon using quantitative PCR. Considering that CD44v splice isoforms show very diverse in HCC1937 cells (Figs. 4D and S3G), which may lead to amplification failure due to multiple PCR peaks during quantitative PCR, we performed the quantitative PCRs of CD44 AS in MDA-MB-231 and HCC1806 cells. Results showed that the CD44v/CD44s ratio was significantly decreased following SRSF3 KD (Fig. 4B, C), but CD44 (total) expression did not change in the MDA-MB-231 and HCC1806 cells (Fig. S3D). The CD44v6-8 and CD44v8-10 expression levels were also detected after KD of SRSF3 and were found to be markedly suppressed in both cell lines (Fig. S3E, F). The qPCR results were validated using semi-quantitative RT-PCR, which showed consistent results (Figs. 4D and S3G).

In addition to loss-of-function, we OE SRSF3 to observe any changes in CD44v (Fig. S3H). Using the same approach as in Fig. 2, we examined the CD44v/CD44s ratio in the MDA-MB-231 and HCC1806 cells. Exogenous expression of SRSF3 induced a significant increase in CD44v abundance in both cell lines (Fig. 4E, F), while CD44 (total) transcription did not change upon SRSF3 OE (Fig. 4G), highlighting the specific regulatory roles of SRSF3 for CD44 AS. The above phenotypes were confirmed via gradient doxycycline induction (Fig. S3I). Of note, increasing the doxycycline dose promoted various CD44v isoforms (Fig. 4H), showing the dose-dependent relationship between SRSF3 and CD44v isoform expression. Those results demonstrated that SRSF3 promoted CD44 variant exon inclusion, and the CD44 variant exons 8 to 10 were significantly regulated under different intervention conditions.

### SRSF3 increases TDP43 abundance by regulating mRNA nonsense-mediated decay (NMD)

**Fig. 5:**
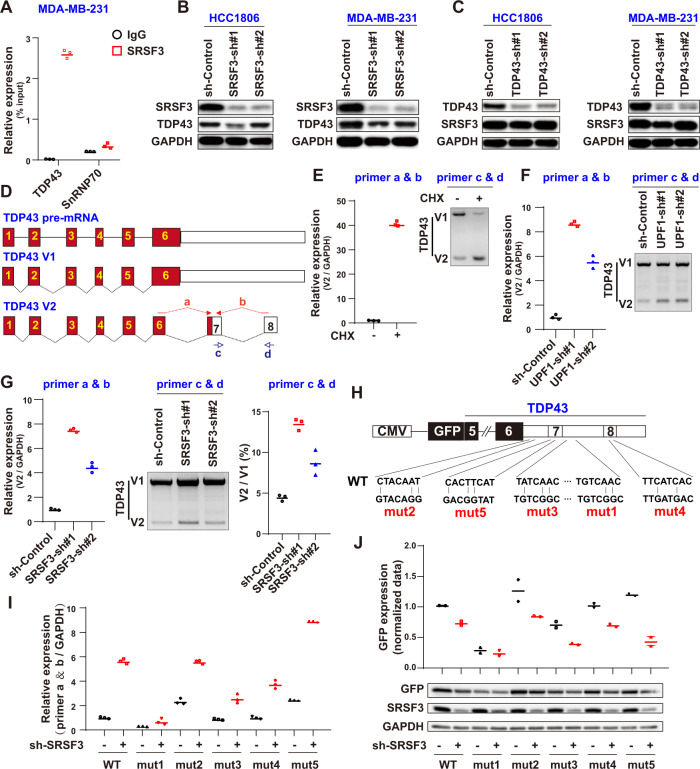
SRSF3 increases abundance of TDP43 by regulating mRNA nonsense-mediated mRNA decay. **A** Binding of TDP43 mRNA to SRSF3 proteins detected by RIPA in MDA-MB-231 cells stably expressing Flag (control) or Flag-SRSF3. **B** Protein levels of TDP43 examined by western blot analysis after SRSF3 KD. **C** Western blot analysis of SRSF3 protein levels after TDP43 KD. **D** Schematic of TDP43 pre-mRNA splicing. qPCR primers a and b; semi-quantitative RT-PCR primers c and d spanning the V2 isoform junction. V2 isoform was detected by indicated primers upon treatment with CHX (**E**), sh-UPF1 (**F**), or sh-SRSF3 (**G**) in MDA-MB231 cells. **H** Schematic of splicing reporter containing GFP gene fused with TDP43 mRNA 3’UTR. Mutations of potential SRSF3 binding sites were introduced as indicated below. **I** qPCR results in MDA-MB-231 cells with transfected GFP reporter. **J** Western blotting of GFP protein upon SRSF3 KD. Data are means ± SD.

To elucidate the relationship between TDP43 and SRSF3 in regulating CD44 AS, we reduced the expression levels of TDP43 and SRSF3 individually and synchronously. However, KD of both TDP43 and SRSF3 did not decrease the CD44v/s ratio compared to KD of TDP43 and SRSF3 individually (data not shown), suggesting that TDP43 and SRSF3 do not regulate CD44 AS in a cooperative way. Surprisingly, based on published CLIP-seq data [43], the SRSF3 protein is known to bind to TDP43 mRNA in mice. Here, using RIP-qPCR with Flag conjugating SRSF3 as a probe (Fig. 5A), we showed that SRSF3 clearly binds to TDP43 mRNA, suggesting that TDP43 may be regulated by SRSF3. To verify this hypothesis, we explored TDP43 expression after SRSF3 KD and found that KD of SRSF3 significantly reduced the TDP43 protein level (Fig. 5B and WB). In contrast, TDP43 KD had a very limited effect on the levels of SRSF3 (Fig. 5C and WB), indicating that TDP43 may be a downstream factor of SRSF3.

For the two main isoforms of TDP43 (V1 and V2), AS occurs in the 3’-untranslated region (3’-UTR), and the unstable V2 isoform undergoes NMD (Fig. 5D) [44]. Based on the binding of SRSF3 to TDP43 pre-mRNA (Fig. 5A), we hypothesized that the SRSF3 protein may bind to the TDP43 3’-UTR to inhibit NMD of the V2 isoform and thereafter increase the abundance of TDP43 V1 mRNA. To verify this hypothesis, the TDP43 V2 mRNA level was detected with specific primers upon SRSF3 KD in the MDA-MB-231 cells (Fig. 5D). Using an NMD inhibitor, cycloheximide, the degradation of the TDP43 V2 isoform was significantly inhibited, confirming the existence of NMD of the TDP43 V2 isoform in breast cancer cells (Fig. 5E). Moreover, depletion of the central component of NMD, i.e., UPF1 [45, 46], enhanced the production of the shorter TDP43 V2 isoform (Fig. 5F), providing another positive control for the NMD of TDP43 V2. Upon KD of SRSF3, the TDP43 V2 isoform increased significantly (Fig. 5G), thus showing the requirement of SRSF3 for TDP43 NMD. To test whether SRSF3 drives splicing of TDP43 pre-mRNA through binding to its 3’UTR, we constructed green fluorescent protein (GFP) reporter plasmids, which contained the *TDP43* 3’UTR region and the 3’UTR region mutated plasmids in the potential SRSF3 binding sites (Fig. 5H). Upon SRSF3 KD, the wild-type mRNA of the shorter 3’UTR form increased (Fig. 5I) and induced downregulation of the GFP protein (Fig. 5J and WB). Mutation of the SRSF3 binding site (mut1) blocked the splicing regulation of SRSF3; however, mut2 partially abolished this effect (Fig. 5I, J). These results indicate that SRSF3 is responsible for TDP43 AS by directly recognizing its binding sites on TDP43.

### SRSF3 enhances CD44v inclusion through regulation of TDP43

**Fig. 6:**
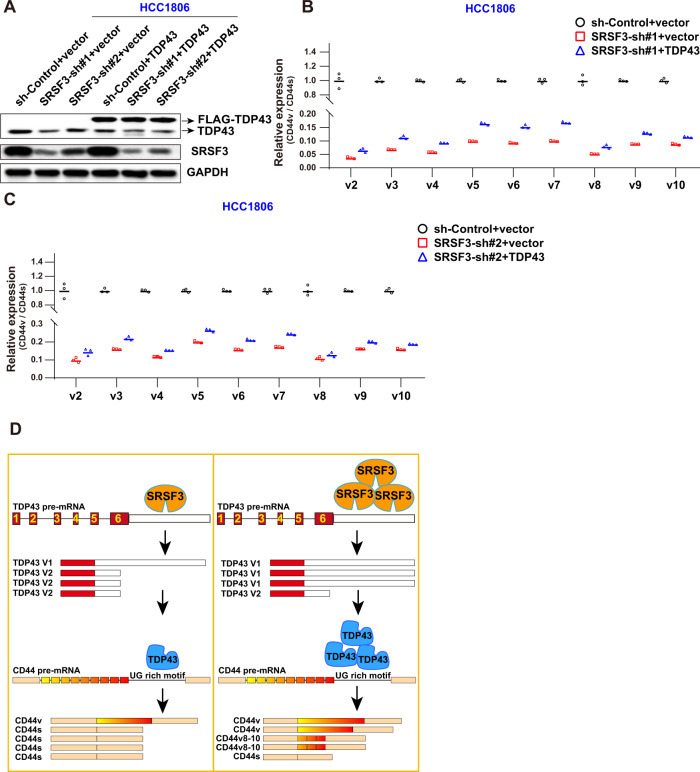
SRSF3 enhances CD44v inclusion through regulation of TDP43. **A** Western blot analysis of SRSF3 KD and TDP43 OE efficiency. **B**, **C** qPCR analysis of CD44v/s following SRSF3 KD, TDP43 OE, or SRSF3 KD/TDP43 OE in HCC1806 cells. **D** Graphical abstract of roles of SRSF3-TDP43 axis in regulating CD44 splicing. High SRSF3 expression increases TDP43 abundance and CD44s expression. After SRSF3 KD, TDP43 levels decrease, reducing TDP43-binding to CD44 pre-mRNA, leading to CD44v to CD44s splicing transformation. Data are means ± SD.

As SRSF3 regulated TDP43 AS directly, we next explored whether SRSF3 promoted CD44v inclusion through TDP43. Our rescue strategy was to overexpress TDP43 ectopically after KD of SRSF3. Results showed that SRSF3 maintained high KD efficiency in both the TDP43 OE and control groups (Fig. 6A and WB). Endogenous TDP43 declined significantly upon SRSF3 KD, while exogenous TDP43 fused with FLAG and showed no obvious change (Fig. 6A and WB). As expected, ectopic expression of TDP43 effectively increased the CD44v/s ratio (Fig. S4A). Furthermore, CD44v expression decreased significantly upon SRSF3 silencing, but the reduction was partially rescued after TDP43 elevation in the MDA-MB-231 (Fig. S4B–D and WB) and HCC1806 cells (Fig. 6B, C). These results indicate that elevation of TDP43 abrogates SRSF3 KD-dependent CD44v reduction in breast cancer cells.

## Discussion

As an important splicing regulator, TDP43 is considered a central pathological protein in neurodegenerative diseases [47]. We also previously identified the regulatory roles of TDP43 in the progression of TNBC [32]. In the current study, based on enrichment of BCSCs in TNBC, TDP43 was also shown to promote BCSC stemness through regulation of CD44 AS, further elucidating the underlying mechanisms of TDP43 in regulating TNBC progression. We also identified another splicing factor, SRSF3, as an upstream regulator of the above mechanism. Notably, SRSF3 increased TDP43 abundance via AS regulation, and further modulated CD44 AS and maintained BCSC stemness (Fig. 6D). Combined with our previous research, we identified the important role of the complex regulatory networks formed by the SRSF3 and TDP43 splicing factors in TNBC progression.

RNA splicing is an indispensable possess in the post-transcriptional regulation of human genes [48, 49], and splicing defects are frequently found in human cancers [50]. Perturbation in the RNA-binding protein (RBP) network is an important aspect of the abnormal changes of AS in cancer [28, 51, 52], relying on an intricate system of competitive and cooperative interactions and mutual control to fine-tune the outcome of AS [53, 54]. Increasing evidence from mammalian genomes indicates that gene splicing and AS are not accomplished by a single splicing factor, but by a network of multiple splicing regulators [55]. For example, the ASD-2 and SUP-12 splicing regulators can bind to the pre-mRNA of nuc-60 and cooperatively promote a switch from isoform UNC-60A to UNC-60B [56]. In addition, SRSF3 and SRSF10 exhibit an antagonistic effect on YTHDC1-dependent mRNA splicing [57], and SRSF1/2 proteins cooperate and compete to regulate splicing [55]. Moreover, splicing factors likely form cross-regulation networks [58] and mutual control to fine-tune the outcome of AS [53, 54]. Evidence shows that different heterogeneous nuclear ribonucleoprotein (hnRNP)-encoding genes contain other binding sites for hnRNPs and regulate RBPs at the splicing level [58]. Therefore, in the process of regulating RNA AS, there is a complex regulatory network among splicing factors. In our previous study, we demonstrated that TDP43 and SRSF3 regulate AS cooperatively [32]. In the current study, we revealed that SRSF3 and TDP43 cooperate by stabilizing the level of TDP43. Therefore, SRSF3 and TDP43 not only exhibit cooperative regulation, but also show a tandem relationship. Our research suggests a potential model for regulating TNBC progression: SRSF3 first stabilizes the abundance of TDP43 mRNA, and thereafter provides enough TDP43 proteins for the cooperative network to regulate various splicing genes, such as *PAR3* and *NUMB*, and thus influence TNBC progression. These results illustrate the complexity of splicing factors.

As the most common CSC marker, CD44 is widely expressed in solid tumors [59], consisting of standard and variant exons. The additional domains encoded by variant exons interact with other cell surface components that underlie the functional diversity between CD44v and CD44s. Compared with the ubiquitous expression of CD44, CD44v is mainly expressed in cancer cells and correlated with CSC stemness, cancer metastasis, invasion, chemoresistance, and poor prognosis [17, 60, 61]. Although CD44 AS has been widely studied, it remains unclear how the CD44 variant exons v8, v9, and v10 are maintained as the predominant AS transcripts [15, 62]. Splicing regulatory elements (SREs) play a pivotal role in accurately and efficiently regulating splicing events [63]. For the SREs of the CD44 variant region, there are two UG-rich motifs within introns 1 and 15, which are well-known TDP43-binding sites [39]. We calculated the UG-rich motif distance to the nearest splicing sites and found that the UG-rich motif within intron 1 was located ~10 kb from exon 2 and 27 kb from exon 1, while the UG-rich motif within intron 15 was located 200 bp from variant exon 10. Our functional assays demonstrated the role of the (UG)_18_ repetitive sequence within intron 15 and confirmed the potential roles of SREs proximal to branch-site residues [22]. In addition, there was a (UG)_3_ extension downstream of variant exon 5, but this motif had no significant effect on AS, indicating that TDP43 may be an important factor in maintaining the inclusion of variant exons close to v10.

For the TDP43 knockdown (KD) assay in Fig. 1E, we prepared four pairs of shRNAs for depletion of TDP43 expression. In our three independent assays, the percentages of cell population with both CD24^low^ and CD44^high^ kept the same deduction trends in KD groups in comparison with their control groups (data not shown). While for single staining by CD24 and CD44 antibodies, we observe large variation among different shRNAs, and speculated that different shRNA targets different isoforms of TDP43, thus varying different downstream effects [64]. Despite of this fact, the decline of CD24^low^CD44^high^ population is consistent treated by four different shRNAs. Besides, our results of mammary sphere and ALDH assay further confirmed that TDP43 KD inhibits BCSC stemness.

Research has demonstrated that SRSF3 can bind to the splicing enhancer during CD44v9 active splicing [41], while other studies have found that SRSF3 promotes CD44 variant exon 8–10 inclusion [42, 65]. To clarify this contradiction, four breast cancer cell lines were used, which consistently showed that SRSF3 elevated CD44v inclusion. To understand the regulatory mechanism of CD44 AS by SRSF3, we also analyzed the potential binding motifs within the CD44 variant exons as SRSF3 is enriched on exons of its target pre-mRNA [66]. We analyzed the CD44 sequence of the variant exon using the online tool SpliceAid [67], which indicated that variant exon 9 has greater binding potential to SRSF3 than the other variant exons (Fig. S4E). These results suggest that SRSF3 can promote CD44v expression by directly binding to CD44 variant exons independent of TDP43. This conjecture was supported by our results showing that TDP43 can partly rescue the reduction in CD44v expression following SRSF3 KD.

Although we found that SRSF3 can modulate CD44 AS through TDP43 at the pre-mRNA level, we cannot rule out the possibility that SRSF3 also cooperates with TDP43 at the protein level. It is possible that AS in some genes may depend on TDP43, while others require both splicing factors for regulation. Moreover, SRSF3 can activate CD44 splicing by binding to the v9 exon splicing enhancer [41] and can specifically mediate the inclusion of v8, v9, and v10 [42, 65], and thus may also regulate CD44 AS by directly binding to CD44 variant exons independent of TDP43. Increasing evidence, including our research, shows that CD44, which is the most common CSC marker, contains various isoforms, and CD44 AS may not be controlled by a single splicing factor or regulatory mode. Instead, CD44 AS may be the result of complex interactions among multiple splicing factors, such as SRSF3 and TDP43. In addition, different isoforms of CD44 regulate cancer progression in many ways and have become an important drug target for tumors. However, more research is needed to clarify the complex splicing regulatory.

SRSF3 regulates cell senescence in many previous reports [68–71]. Tang et al. reported that downregulation of SRSF3 induces p53β, an alternatively spliced isoform of p53 that promotes cellular senescence. Shen et al. also found that SRSF3-depletion also induced senescence-related phenotypes in both human and mouse cells. This result further indicated that SRSF3 may regulate breast cancer cell-renewal through different pathway, such as cellular senescence.

## Materials and methods

### Cell culture

All cell lines were obtained from the American Type Culture Collection (USA) and were maintained under standard culture conditions (37 °C, 5% CO_2_) in culture medium with 1% penicillin/streptomycin solution. Human breast cancer cell lines HCC1806, HCC1937, and MDA-MB-468 were grown in Roswell Park Memorial Institute 1640 medium (11875093, Gibco) with 4 mM L-glutamine and 10% fetal bovine serum (FBS). The MDA-MB231 cells were cultured in Dulbecco’s Modified Eagle Medium/Nutrient Mixture F-12 (DMEM/F-12, 11330032, Gibco) with 4 mM L-glutamine and 10% FBS. The HEK293T cells were cultured in DMEM (11965092, Gibco) with 4 mM L-glutamine, 4.5 g/l glucose, 1 mM sodium pyruvate and 10% FBS.

### Knockdown and overexpression

The shRNAs were constructed using the PLKO.1-based lentiviral technique and were transfected with psPAX2 and pMD2.G (4:3:1) using transfection agent Polyethylenimine (23966, Polyscience) into 293T cells to produce lentiviral particles. The lentiviral particles were then infected into target cells with polybrene, and fresh medium was added after 24 h. After 96 h of infection, the KD efficiency was verified by real-time qPCR and western blot analysis without puromycin selection. For overexpression, full-length TDP43 (27470, Addgene) and SRSF3 (46736, Addgene) vectors were subcloned into the pTRIPZ lentiviral expression vector. Lentiviral particles were produced by co-transfection of 293T cells with pTRIPZ-TDP43 or pTRIPZ -SRSF3, psPAX2, and pMD2.G plasmids and the infected target cells were selected with puromycin to establish stable cell lines.

### Mammosphere assay

The HCC1806 and MDA-MB-231 cells were plated in ultra-low attachment 96-well plates with EpiCult-B Basal Medium (Human) (Stem Cell Technologies, BC, Canada) and EpiCult-B Proliferation Supplement (Human) (Stem Cell Technologies, BC, Canada) with hydrocortisone (H811182, Macklin, China) and heparin (Stem Cell Technologies, BC, Canada). The mammosphere formation efficiency was calculated after 10–14 days.

### Flow cytometry

To examine the ratio of BCSCs, cells were stained with antibodies, including fluorescein isothiocyanate-conjugated anti-CD44 (BD Pharmingen, 555478 for all the CD44 isoforms [10]) and phycoerythrin-conjugated anti-CD24 antibodies (555428, BD Pharmingen) according to the manufacturer’s instructions. ALDH enzymatic activity was assessed using an ALDEFLUOR kit (1700, Stem Cell Technologies) according to the provided manual.

### RNA extraction, qPCR, and semi-quantitative RT-PCR

Total RNA from cells was extracted using TRIzol reagent (Invitrogen, USA) and reverse-transcribed using HiScript III RT SuperMix for qPCR (+gDNA wiper) (Vazyme, China) according to the manufacturers’ instructions. The cDNA was then used for RT-PCR or qRT-PCR. RT-PCR was conducted using 2× Goldstar Best Master Mix (CWBIO, China), and qRT-PCR was performed using iTaq™ universal SYBR® Green (Bio-Rad, USA). Primers are listed in Table S1.

### Western blotting

Cells were washed with phosphate-buffered saline and lysed in Radio-Immunoprecipitation Assay lysis buffer (R21237, Shanghai Yuanye Biotechnology) with a protease inhibitor mixture (B14001, BioTools, USA) on ice for 30 min. The eluents were analyzed by western blotting. Lysis samples were separated by sodium dodecylsulphate-polyacrylamide gel electrophoresis, transferred onto polyvinylidene fluoride membranes, and blocked with 5% nonfat dried milk for 1 h. The membranes were then incubated with the indicated primary antibodies at 4 °C overnight and with horseradish peroxidase-HRP-conjugated secondary antibodies for 1 h, then detected using a chemiluminescent HRP substrate (Millipore, USA). The antibodies used for immunoblotting included: TDP43 (ab109535, Abcam, USA, 1:1000), SRSF3 (ab198291, Abcam, USA, 1:1000), c-Myc (A19032, ABclonal, China, 1:1000), Sox9 (D8G8H, CST, USA, 1:1000), GFP (600-101-215, Rockland, USA, 1:1000), and GAPDH (AP0063, Bioworld, China, 1:5000).

### RNA immunoprecipitation

An EZ-Magna RIP kit (17-701, Millipore, USA) was used for RNA immunoprecipitation assay according to the manufacturer’s instructions. The HCC1806 and MDA-MB-231 cells were collected and lysed in RIP lysis buffer. The cell lysate was then immunoprecipitated with TDP43 (1:10) (10782-2-AP, Proteintech, USA), Flag (1:20) (14793S, CST, USA), or IgG 5 μg (17-701, Millipore, USA) and protein A/G magnetic beads for 6–8 h at 4 °C. The beads were washed with RIP buffer to discard unbound material. RNA was purified and qPCR analysis was performed. The related primers are listed in Table S1.

### Statistical analysis

Unless otherwise indicated, experiments were three times biological repeats, and data shown are representative. To test the significance, Student’s *t* test (two-sided) was used for two-sample comparisons. One-way or two-way analysis of variance was performed for multiple-sample comparisons.

## Supplementary information

Supplementary Information

figure-S1

figure-S2

figure-S3

figure-S4

Fig. WB

aj-checklist

## Data Availability

All data needed to evaluate the conclusions in the paper are present in the paper. Additional data related to this paper may be requested from the corresponding author.
