# Supplementary material for: TDP43 promotes stemness of breast cancer stem cells through CD44 variant splicing isoforms
Source: Cell Death Dis. 2022 May 3;13(5):428. doi: 10.1038/s41419-022-04867-w (PMC9065105; doi:10.1038/s41419-022-04867-w)
Supplement: Supplementary file 1 — Supplementary Information [file 41419_2022_4867_MOESM1_ESM.docx]

Supplementary Table S1. Primers and shRNA sequences used in this paper

| Primers | Sequence |
| --- | --- |
| Sh-TDP43-1F | CCGGGCTCTAATTCTGGTGCAGCAACTCGAG  TTGCTGCACCAGAATTAGAGCTTTTTG |
| Sh-TDP43-1 R | AATTCAAAAAGCTCTAATTCTGGTGCAGCAACTCGAG  TTGCTGCACCAGAATTAGAGC |
| sh-TDP43-2 F | CCGGGGTGCTGCTCTCCACGGTTACCTCGAG  GTAACCGTGGAGAGCAGCACCTTTTTG |
| sh-TDP43-2 R | AATTCAAAAAGGTGCTGCTCTCCACGGTTACCTCGAG  GTAACCGTGGAGAGCAGCACC |
| sh-SRSF3-1 F | CCGGCAGTGACACAAAGGTGTAATTCTCGAG  AATTACACCTTTGTGTCACTGTTTTTG |
| sh-SRSF3-1 R | AATTCAAAAACAGTGACACAAAGGTGTAATTCTCGAG  AATTACACCTTTGTGTCACTG |
| sh-SRSF3-2 F | CCGGGCTAGATGGAAGAACACTATGCTCGAG  CATAGTGTTCTTCCATCTAGCTTTTTG |
| sh-SRSF3-2 R | AATTCAAAAAGCTAGATGGAAGAACACTATGCTCGAG  CATAGTGTTCTTCCATCTAGC |
| CD44v2-F | GCAACCAAGAGGCAAGAAA |
| CD44v2-R | CAGCCATTTGTGTTGTTGTGTG |
| CD44v3-F | CGTCTTCAAATACCATCTCAGC |
| CD44v3-R | CAATGCCTGATCCAGAAAAAC |
| CD44v4-F | TGACCACACAAAACAGAACC |
| CD44v4-R | GTTGTCTGAAGTAGCACTTCC |
| CD44v5-F | GAAATGGCACCACTGCTTATG |
| CD44v5-R | GTCTCTTCTTCCTCATGATGCT |
| CD44v6-F | AGGAACAGTGGTTTGGCAAC |
| CD44v6-R | CGAATGGGAGTCTTCTCTGG |
| CD44v7-F | TCAGCTCATACCAGCCATCC |
| CD44v7-R | TCCTTCTTCCTGCTTGATGAC |
| CD44v8-F | TCAGCCTACTGCAAATCCAA |
| CD44v8-R | GAGGTCCTGTCCTGTCCAAA |
| CD44v9-F | AGCAGAGTAATTCTCAGAGCTTC |
| CD44v9-R | TCAGAGTAGAAGTTGTTGGATGG |
| CD44v10-F | GGAATGATGTCACAGGTGGA |
| CD44v10-R | AGGTCACTGGGATGAAGGTC |
| CD44V6-8 F | GCCAAACACCCAAAGAAGACTC |
| CD44V6-8 R | AGGCTGAAGCGTTATACTATGAC |
| CD44V8-10-F | TCCCTGCTACCAATATGGACTC |
| CD44V8-10-R | CAGAGTAGAAGTTGTTGGATGGTC |
| CD44-Total-F | CGCAGATCGATTTGAATATAACC |
| CD44-Total-R | CCGATGCTCAGAGCTTTCTC |
| CD44 RT-PCR-F | CAGCACTTCAGGAGGTTACAT |
| CD44 RT-PCR-R | TTCAGATCCATGAGTGGTATGGG |
| CD44 v2-F | GATGAGCACTAGTGCTACAG |
| CD44 v3-F | ACGTCTTCAAATACCATCTC |
| CD44 v4-F | TCAACCACACCACGGGCTTT |
| CD44 v5-F | GTAGACAGAAATGGCACCAC |
| CD44 v6-F | GAGGCAACTCCTAGTAGTAC |
| CD44 v7-F | CAGCCTCAGCTCATACCAGC |
| CD44 v8-F | TCCAGTCATAGTACAACGCT |
| CD44 v9-F | CAGAGCTTCTCTACATCACA |
| CD44 v10-F | GGTGGAAGAAGAGACCCAAA |
| CD44 v-R | CCAAGATGATCAGCCATTCTGG |
| endo-TDP43-F | CCTTGCGTTCATAGCGTTGATAC |
| endo-TDP43-R | TGCCATAGGAATACTGTCTACATGC |
| exo-TDP43-F | GGGTAACCGAAGATGAGAACG |
| exo-TDP43-R | CTGGGCTGTAACCGTGGAG |
| homo-SRSF3-1F | TGGCAACAAGACGGAATTGGA |
| homo-SRSF3-1R  primer a  primer b  primer c  primer d | CAAAGCCGGGTGGGTTTCTA  AAAGAAGTGGAAGATTTGGTGTT  TCTTTGCATTCAGGGCGTC  TCGTCATCACGCATCACAGG  TCTGGGTATCAGGTGTCAATGC |

**Figure legends**

**Fig. S1**

(A) CD44 total, (B) CD44v6-8 and (C) CD44v8-10 mRNA expression in MDA-MB-231 and HCC1806 cells after KD TDP43. (D) TDP43 OE efficiency in MDA-MB-231 and HCC1806 cells. (F) CD44 total mRNA expression in MDA-MB-231 and HCC1806 cells after TDP43 OE. (G) TDP43 expression in MDA-MB-231 cells with transfected inducible TDP43 expression vector.

**Fig. S2**

ALDH analysis upon (A) SRSF3 KD, (B) SRSF3 OE in HCC1806 and MDA-MB-231 cells. (C) Western blotting analysis of SRSF3 protein levels in MCF7 cells upon SRSF3-OE. (D) Flow cytometry analysis of ratios of CD44 ^+^/CD24 ^−/low^ population after SRSF3 OE in MCF7 cells. (E) Calculation of number of tumorsphere in MCF7 cells upon SRSF3 OE.

**Fig. S3**

Knockdown efficiency of SRSF3 in (A) protein levels in HCC1806 cells and (B) RNA levels in HCC1937 cells. (C) Semi-quantitative RT-PCR (CD44 RT-PCR) of CD44 isoforms after KD SRSF3 in MDA-MB-468 cells. (D) CD44 total, (E) CD44v6-8 and (F) CD44v8-10 mRNA expression in MDA-MB-231 and HCC1806 cells after SRSF3 KD. (G) CD44v5 various PCR bands amplified using v5-F and v-R primers (indicated in Figure 2A) were cut and purified for DNA sequencing. The sequencing results showed that there are at least three major CD44 variants (arrow) containing V5 exon in HCC1937 cells. (H) SRSF3 OE efficiency in MDA-MB-231 and HCC1806 cells. (I) SRSF3 expression in MDA-MB-231 cells with transfected inducible SRSF3 expression vector.

**Fig. S4**

(A) qPCR analysis of changes in CD44 v/s with TDP43 OE in MDA-MB-231 cells. (B) Western blot analysis of SRSF3 KD and TDP43 OE efficiency in MDA-MB-231 cells. (C and D) qPCR analysis of CD44v/s following SRSF3 KD, TDP43 OE, or SRSF3 KD/TDP43 OE in MDA-MB-231 cells. (E) SRSF3 bind motif within CD44 the variant exon by using the online tool SpliceAid.
