# Supplementary figures and images for: TDP43 promotes stemness of breast cancer stem cells through CD44 variant splicing isoforms
Source: Cell Death Dis. 2022 May 3;13(5):428. doi: 10.1038/s41419-022-04867-w (PMC9065105; doi:10.1038/s41419-022-04867-w)

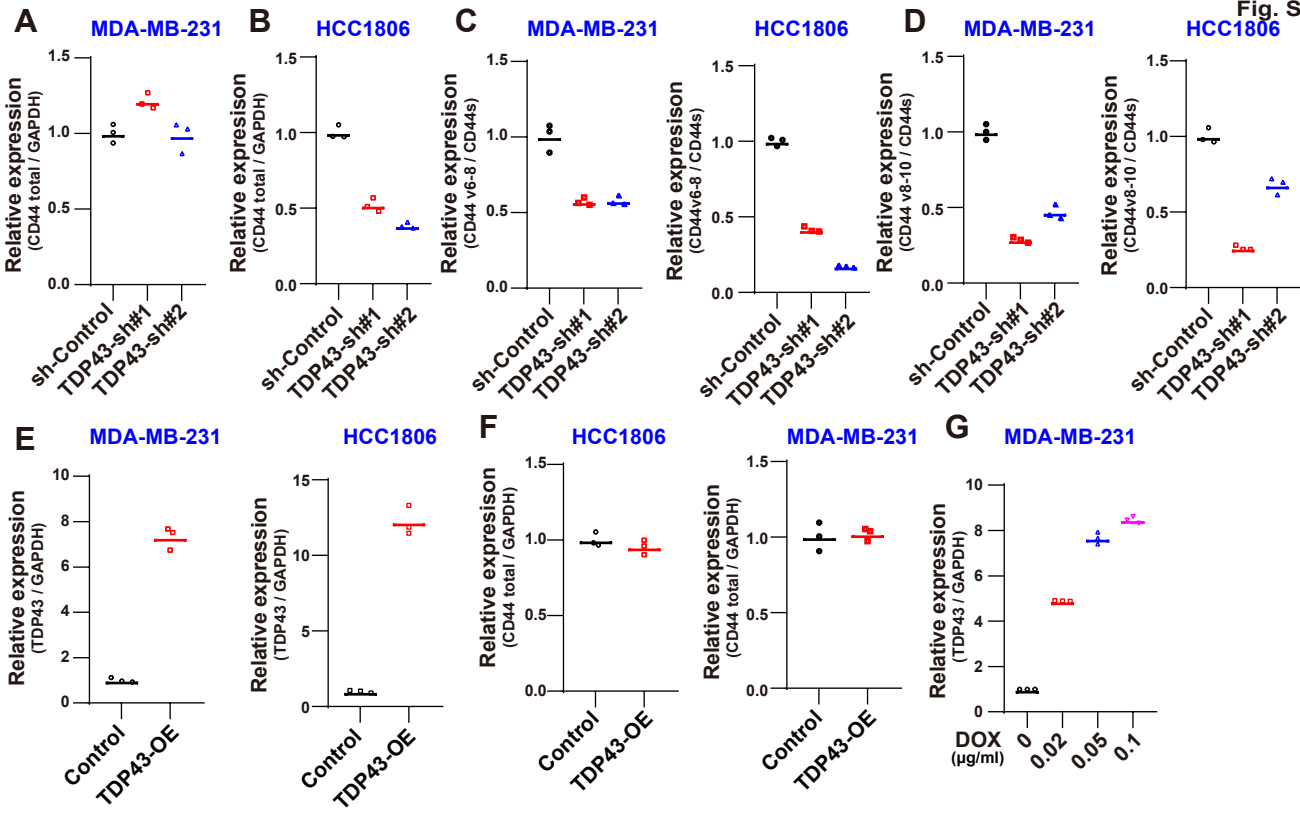

Supplement: Supplementary file 2 — figure-S1 [file 41419_2022_4867_MOESM2_ESM.pdf]

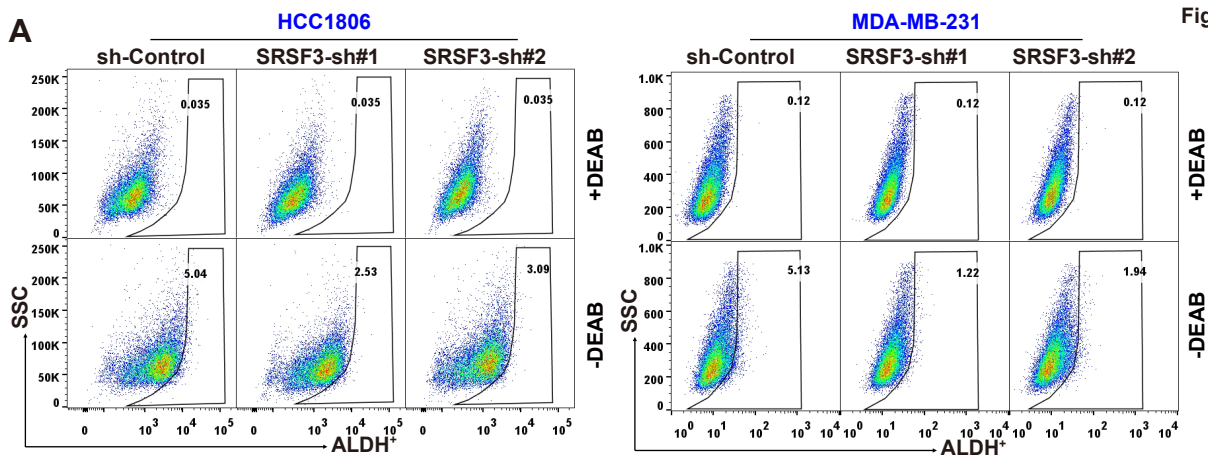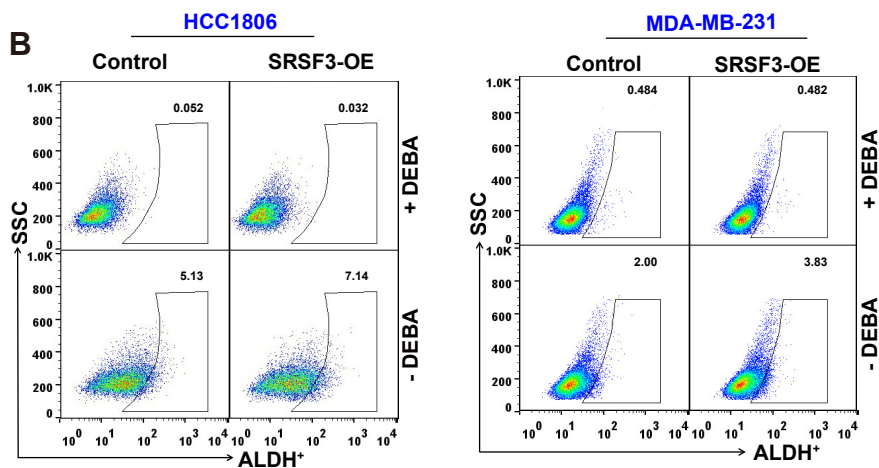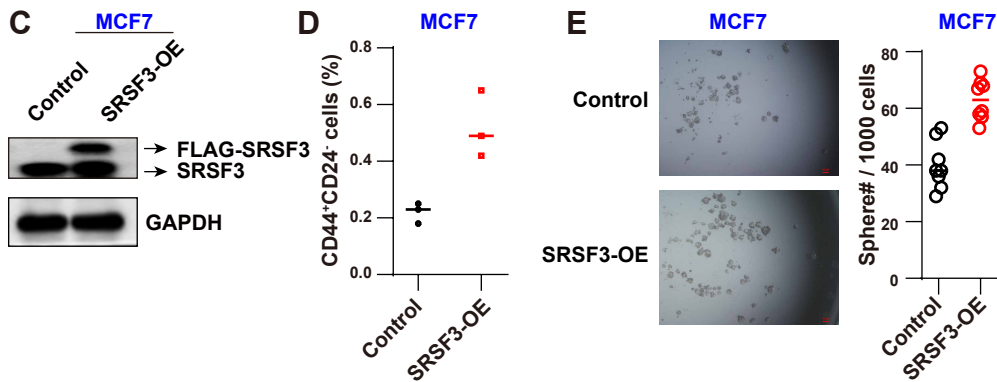

Supplement: Supplementary file 3 — figure-S2 [file 41419_2022_4867_MOESM3_ESM.pdf]

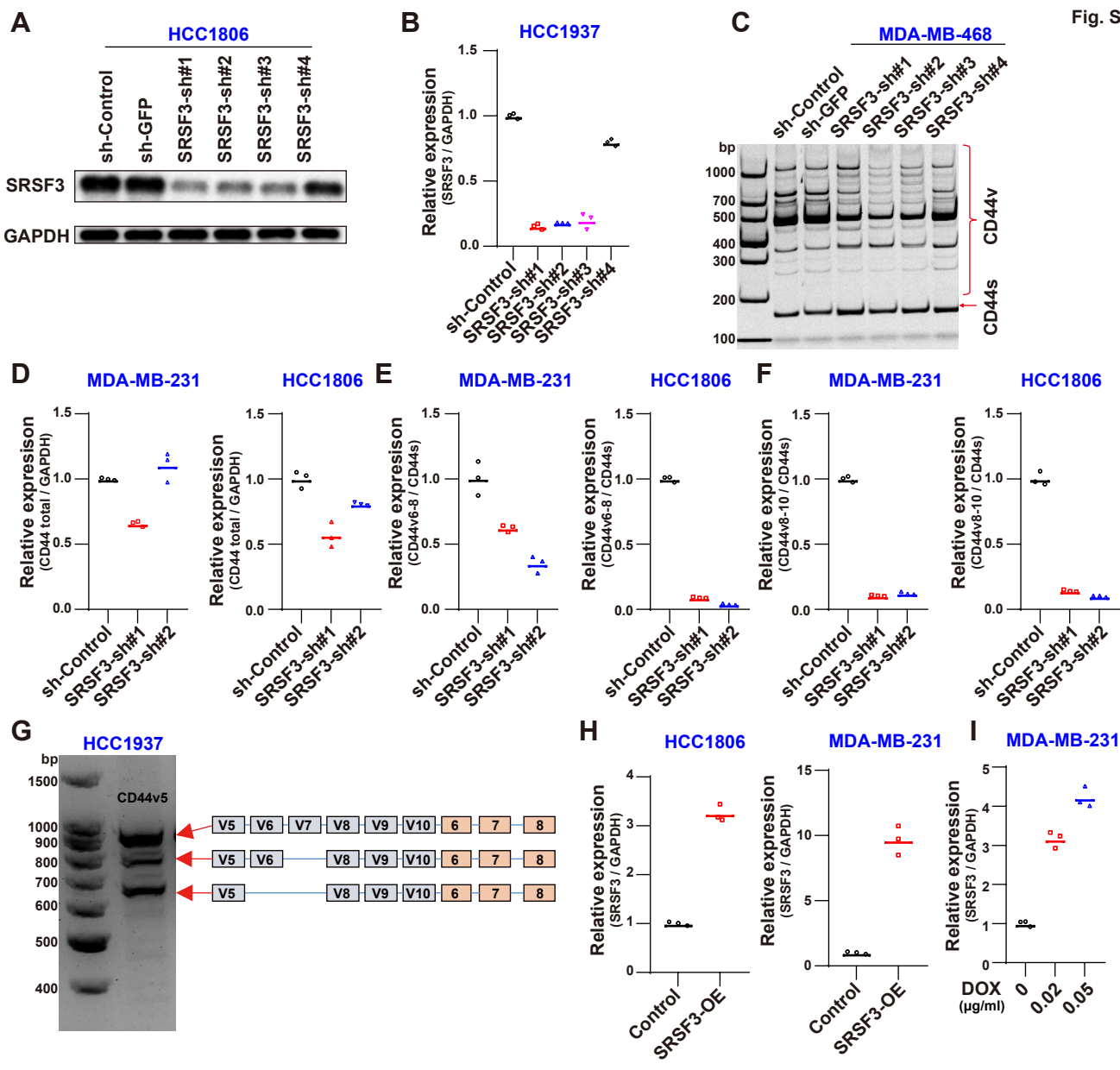

Supplement: Supplementary file 4 — figure-S3 [file 41419_2022_4867_MOESM4_ESM.pdf]

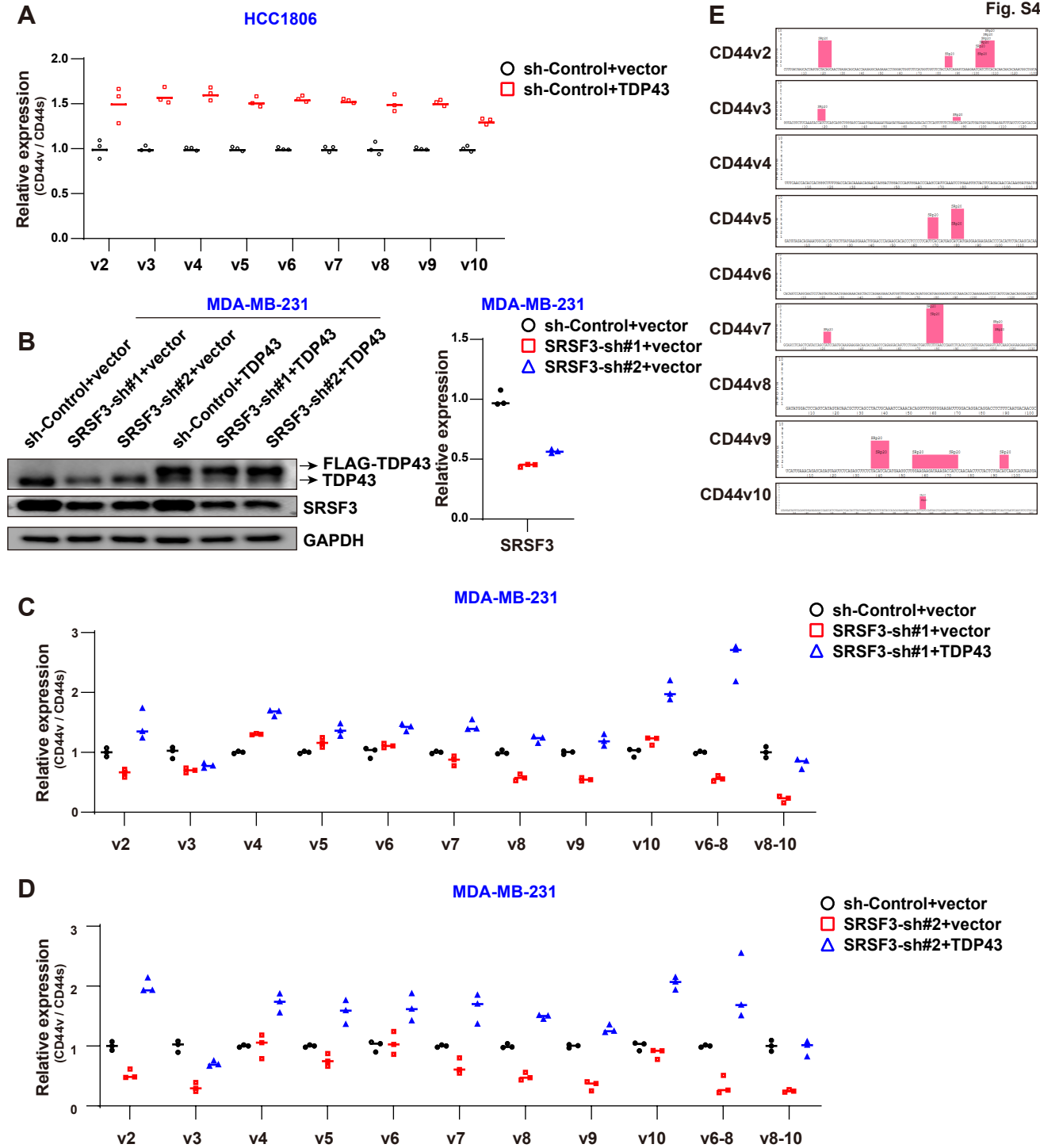

Supplement: Supplementary file 5 — figure-S4 [file 41419_2022_4867_MOESM5_ESM.pdf]

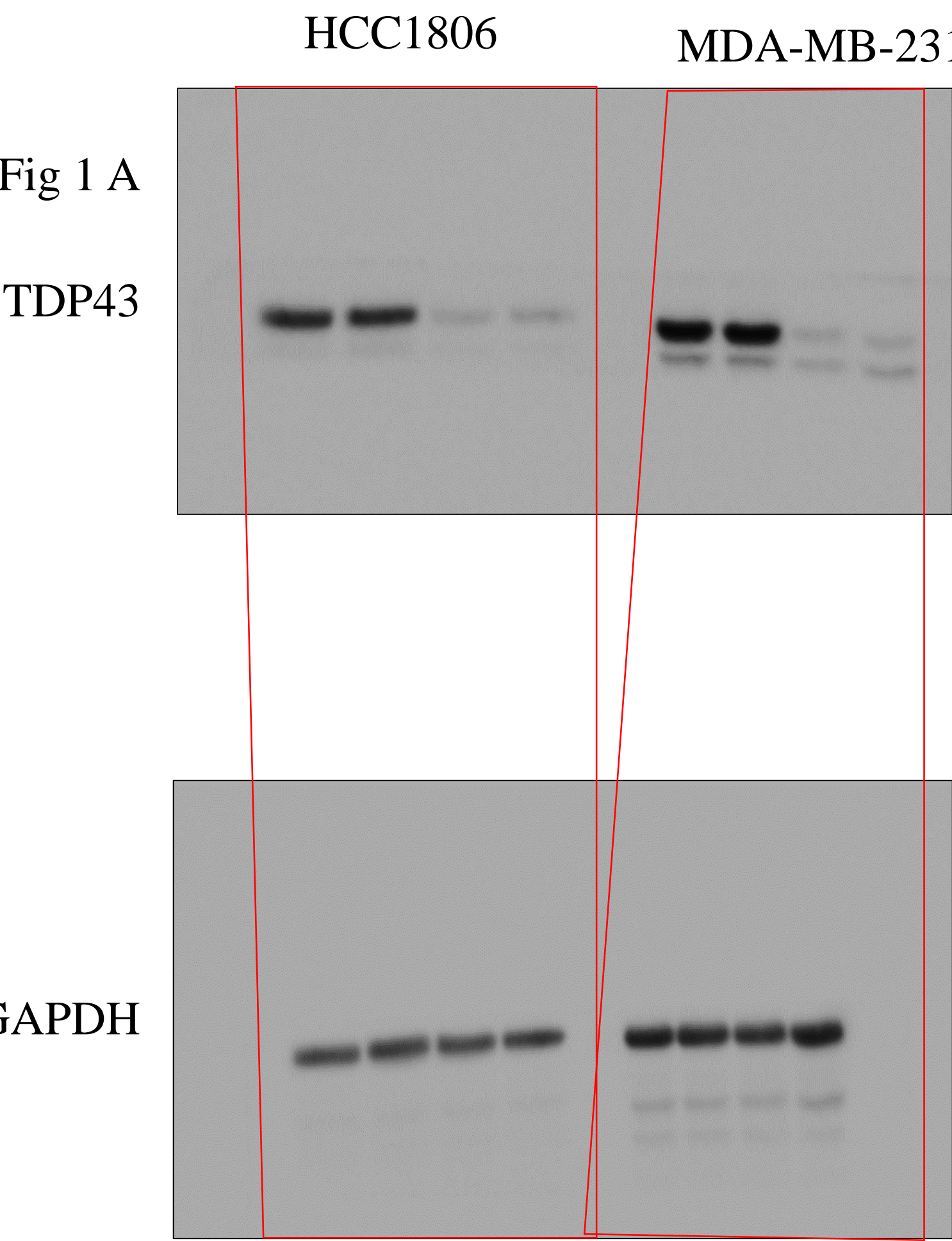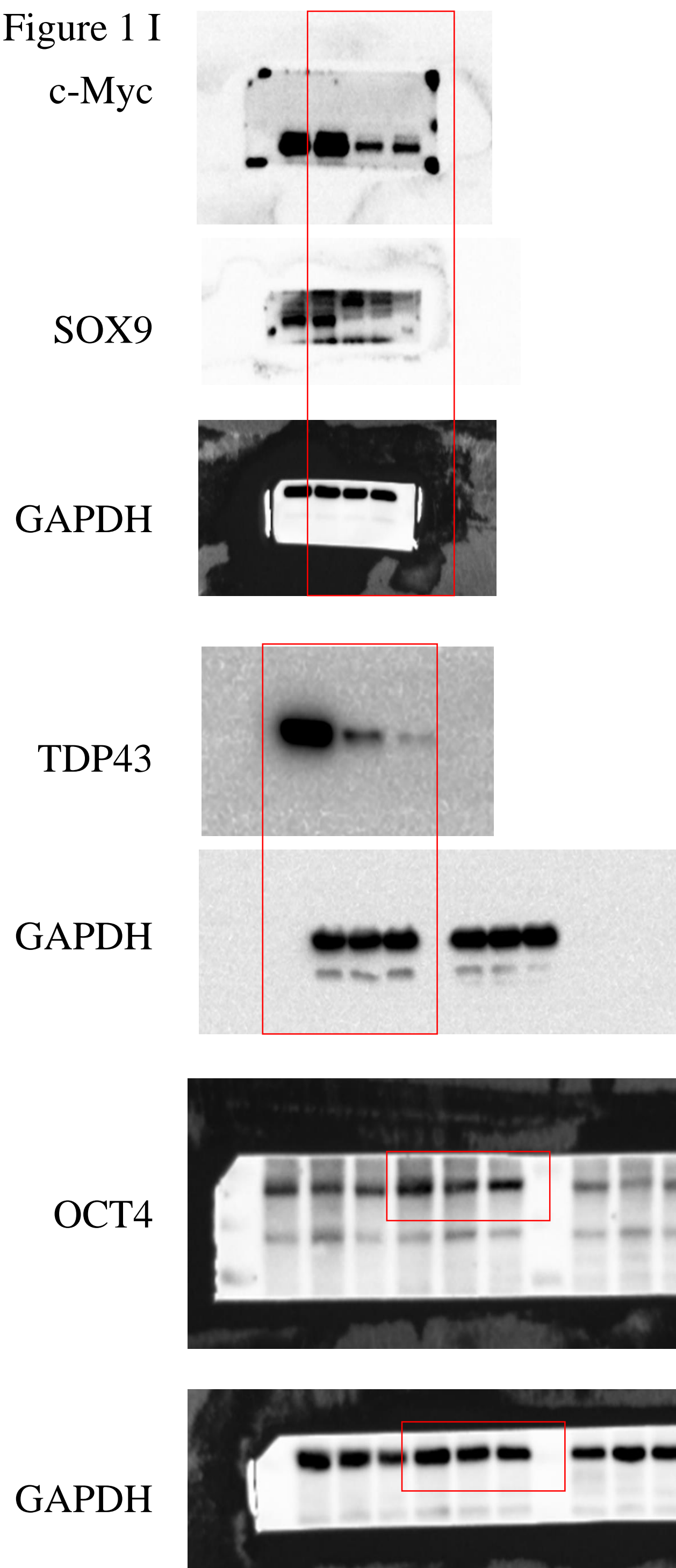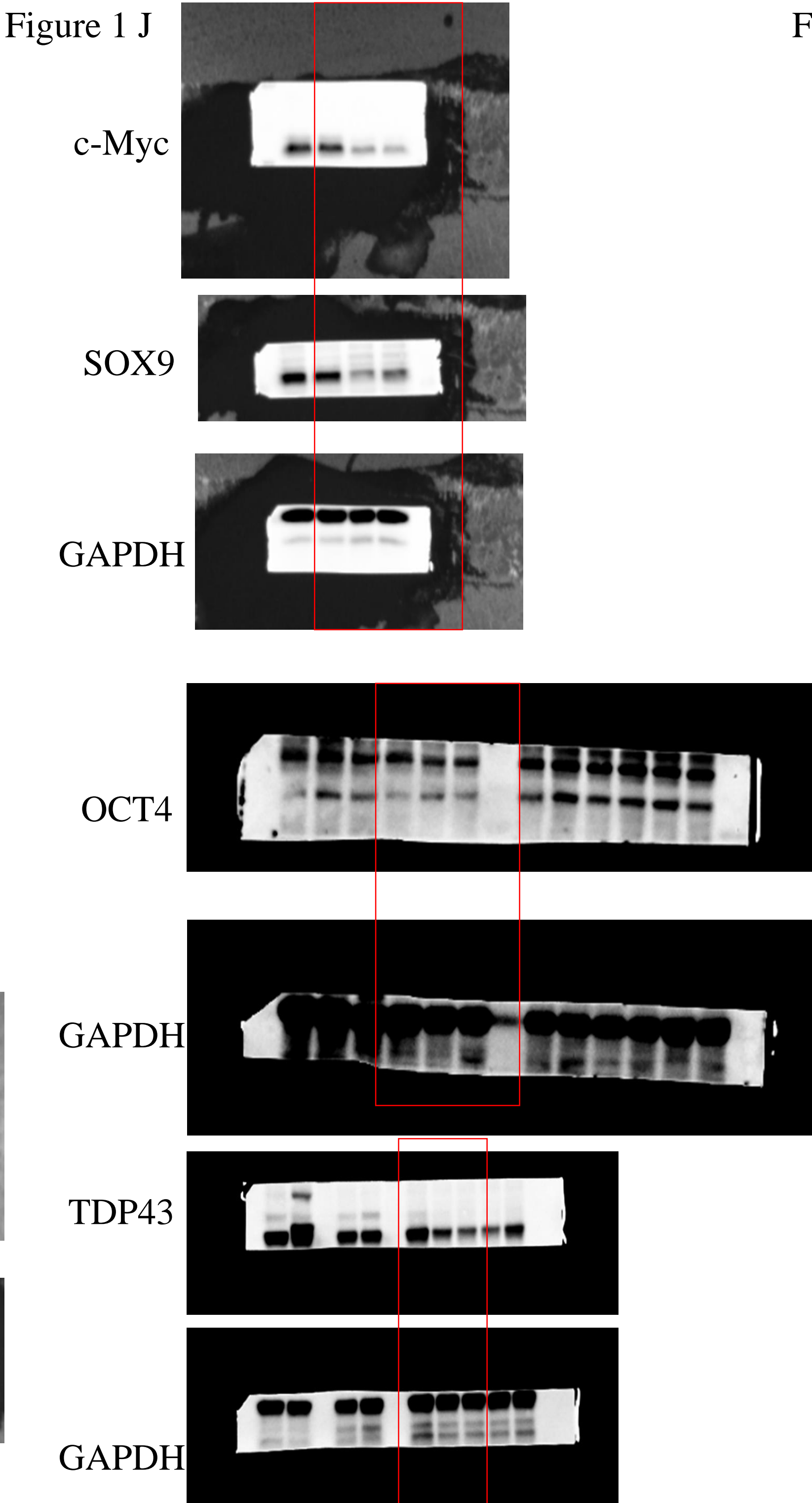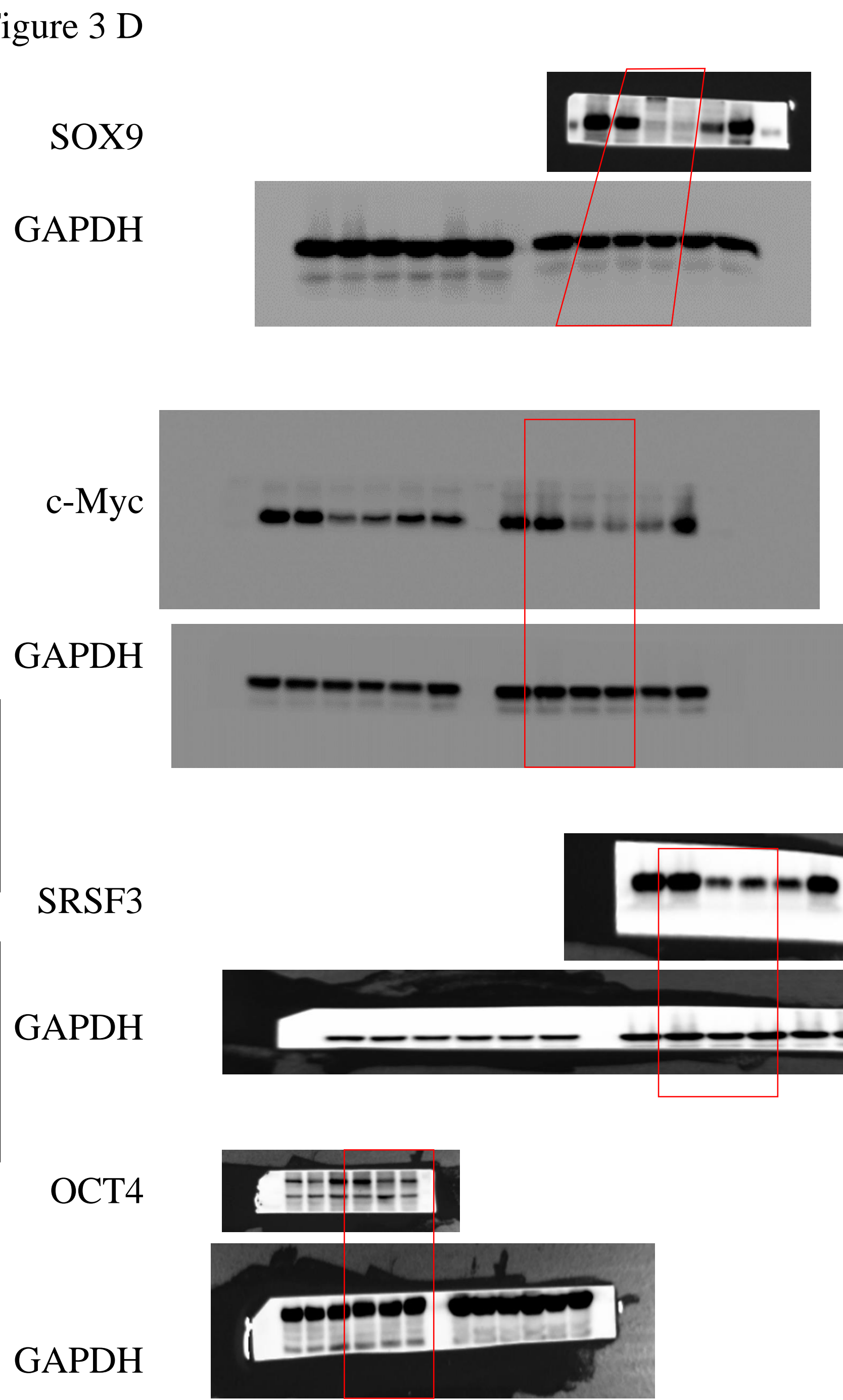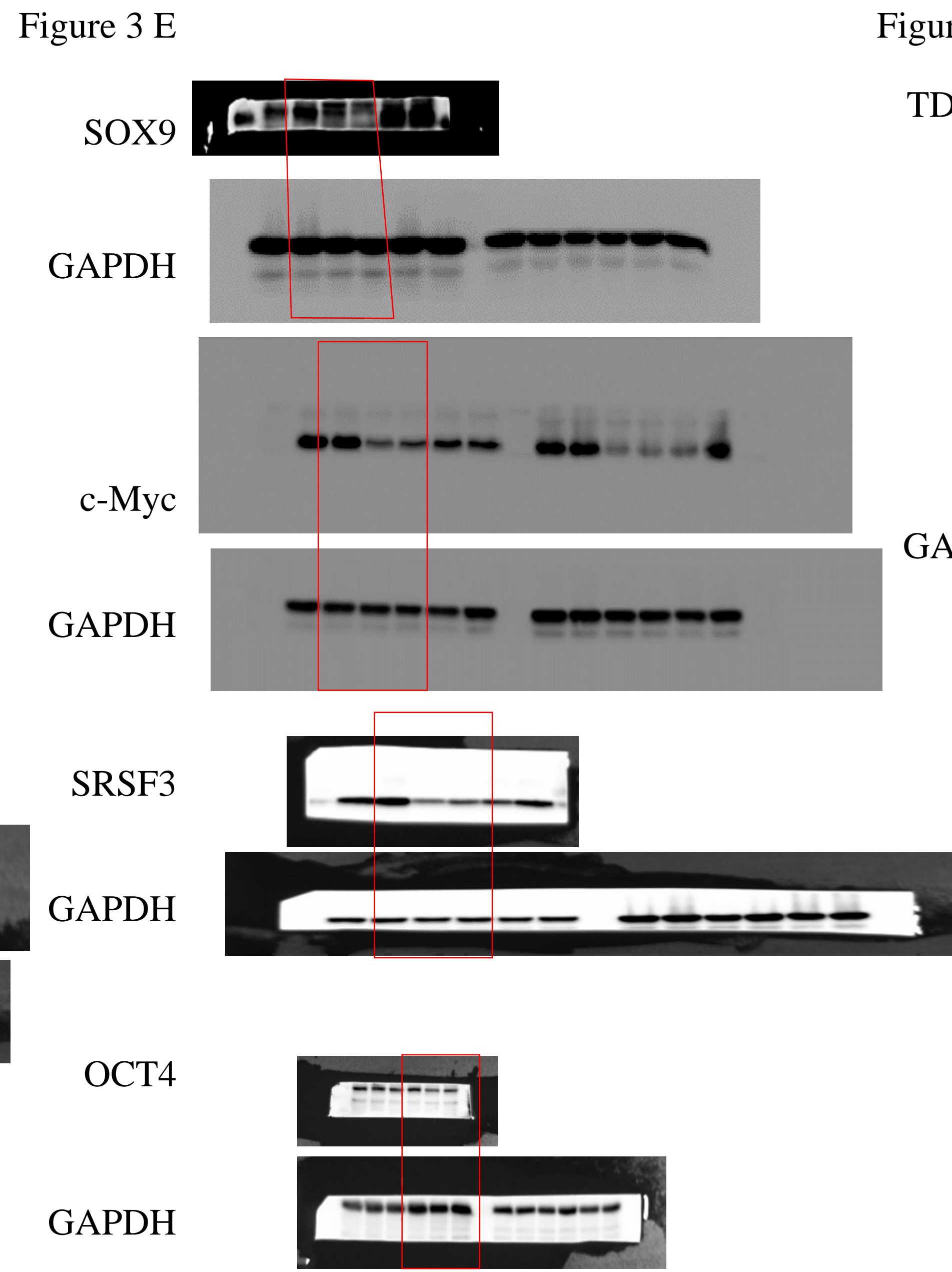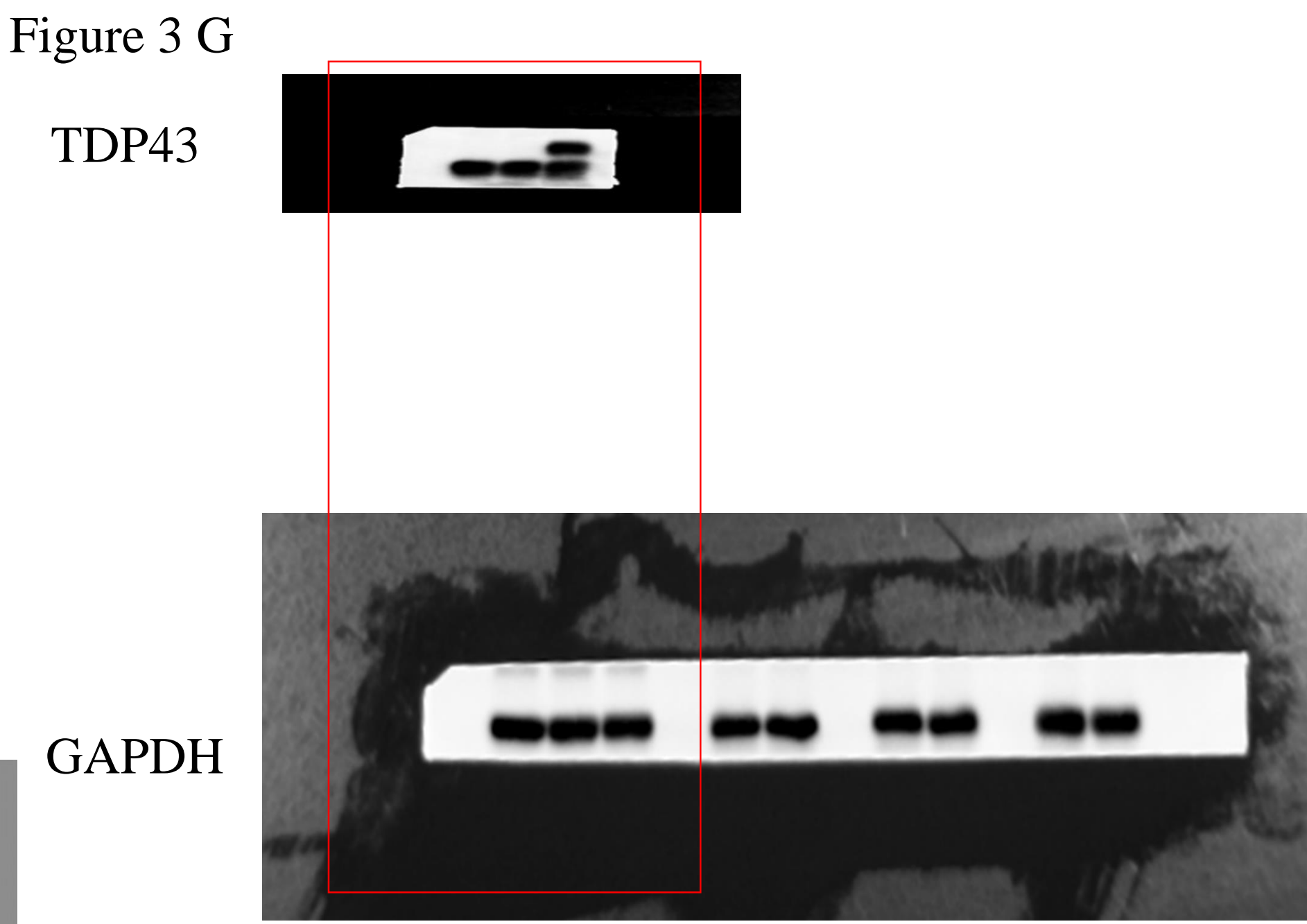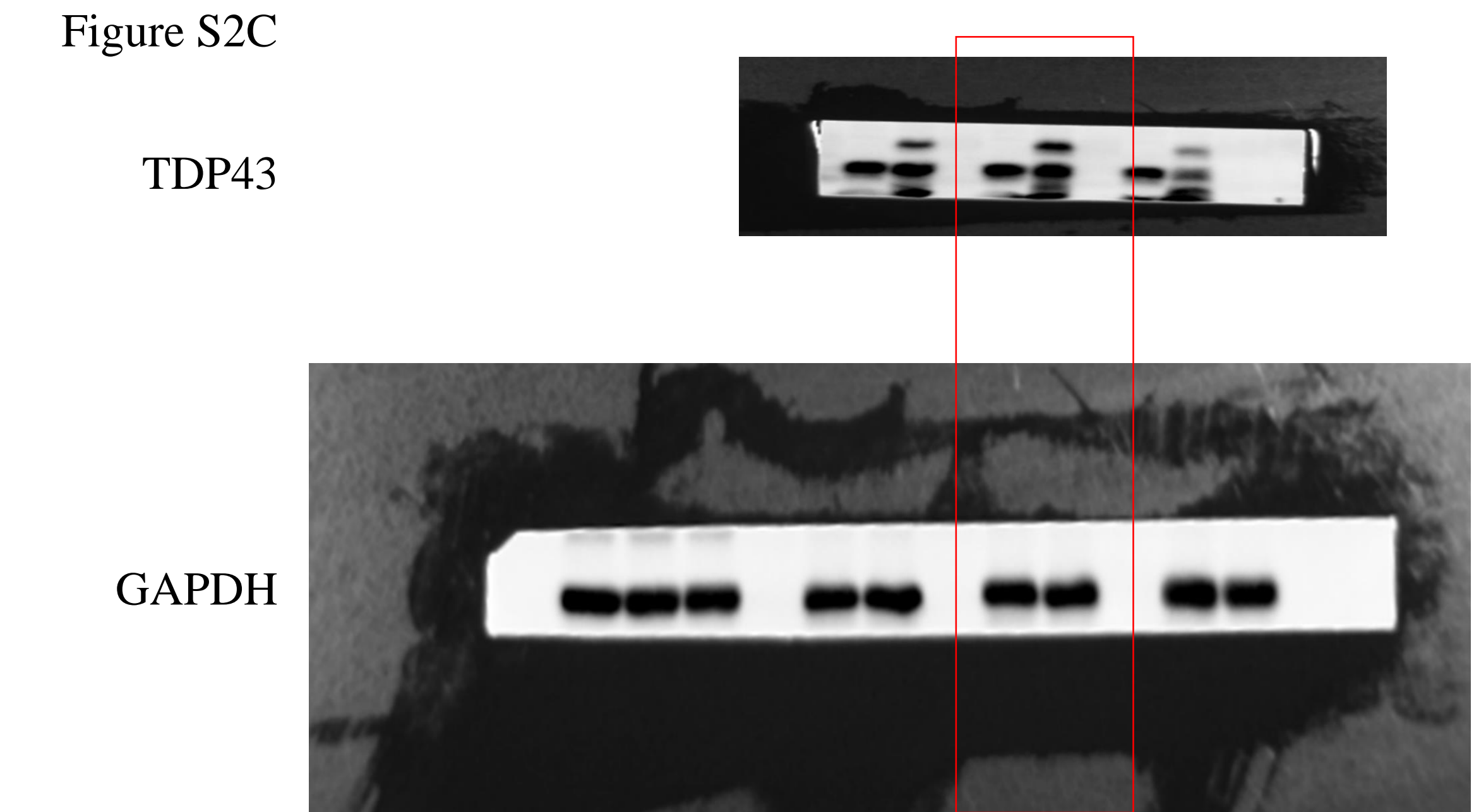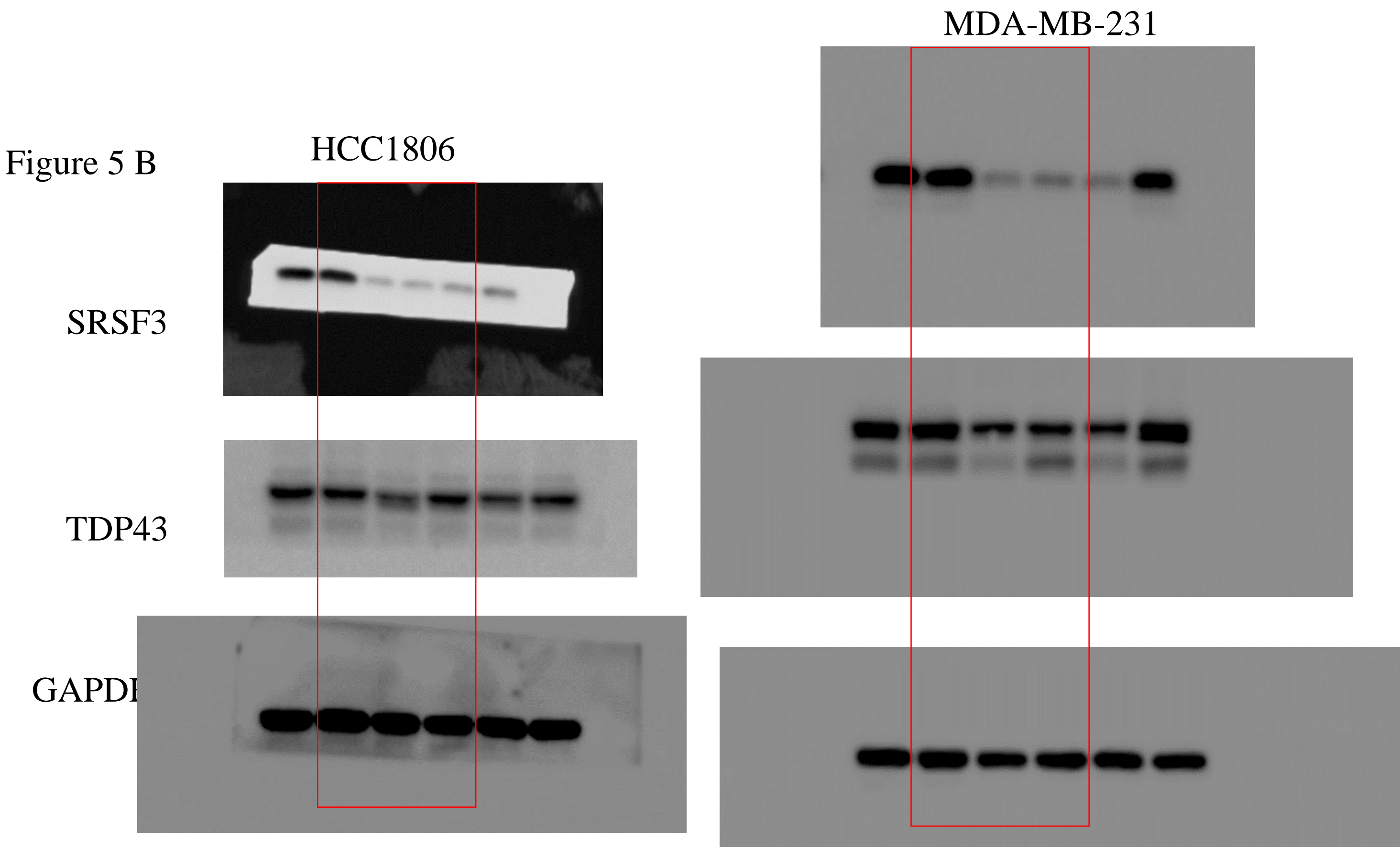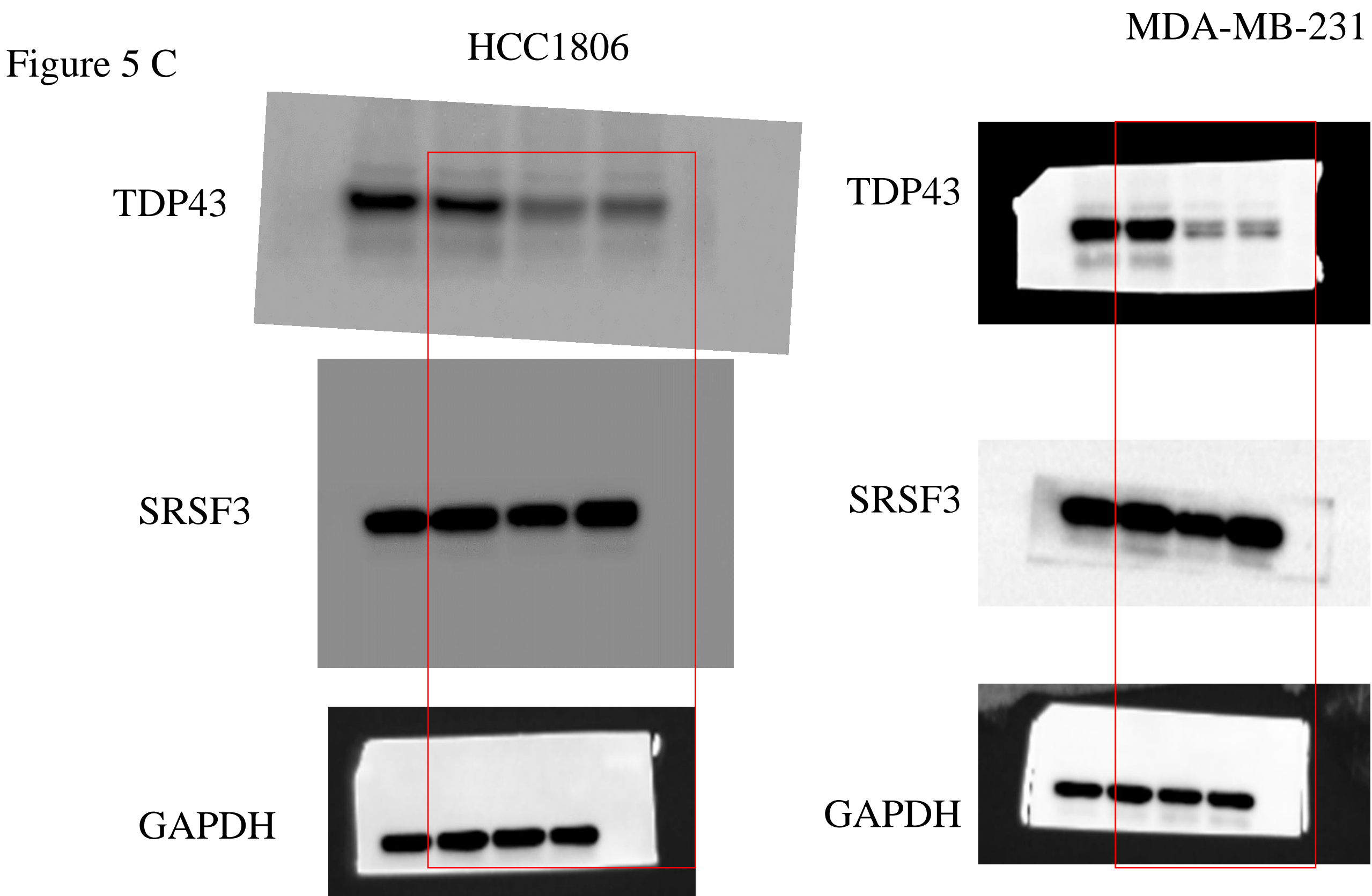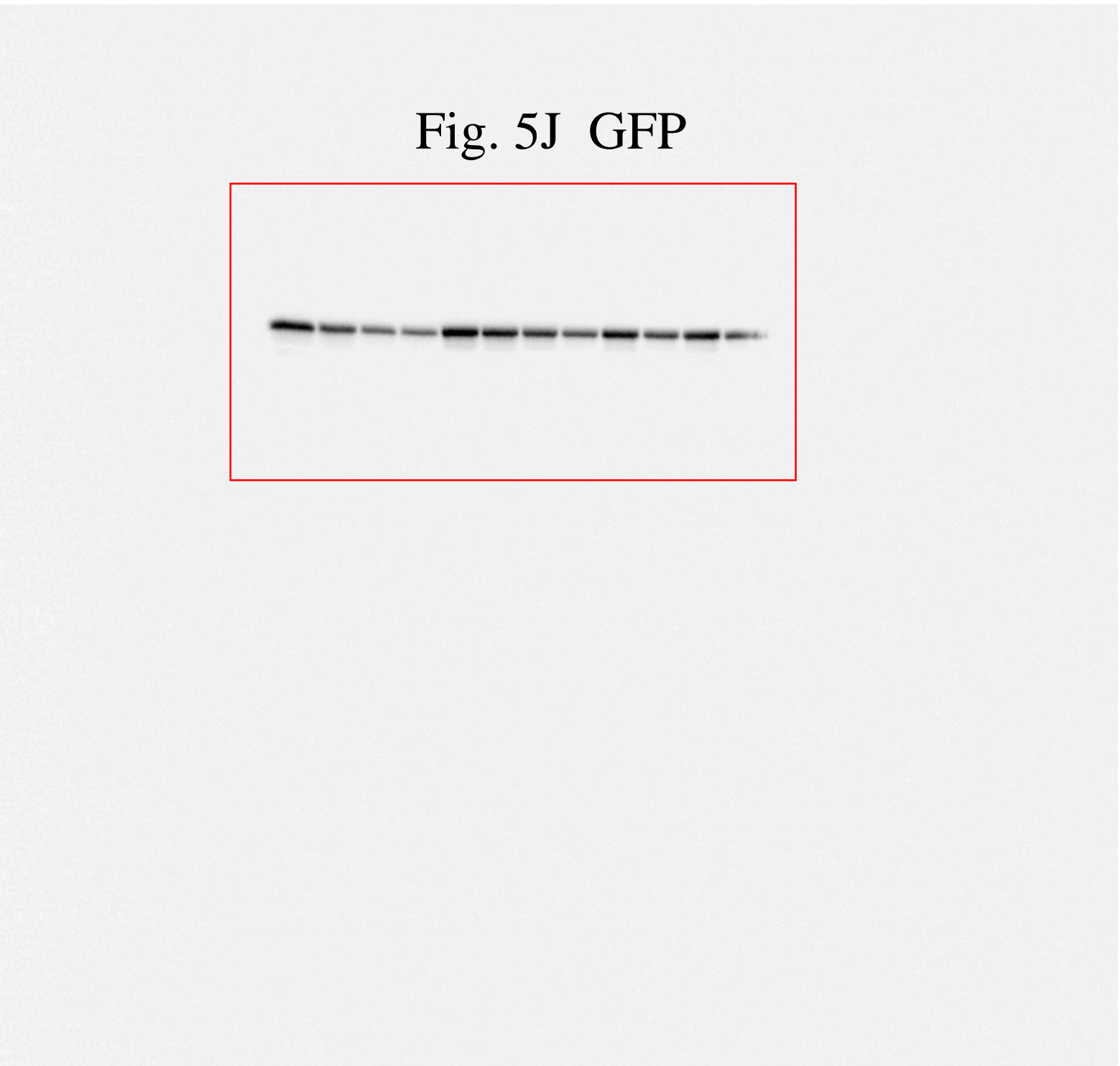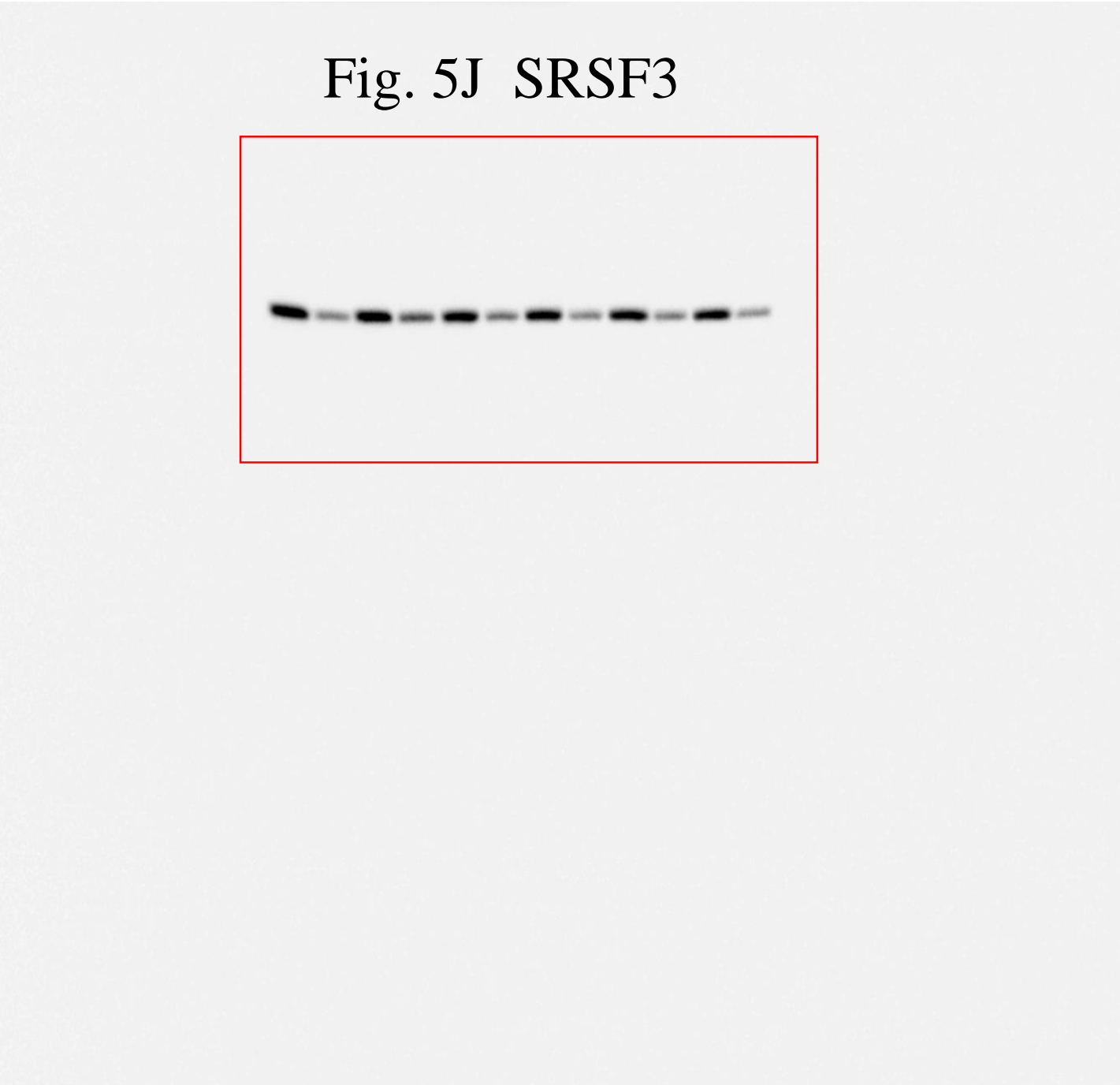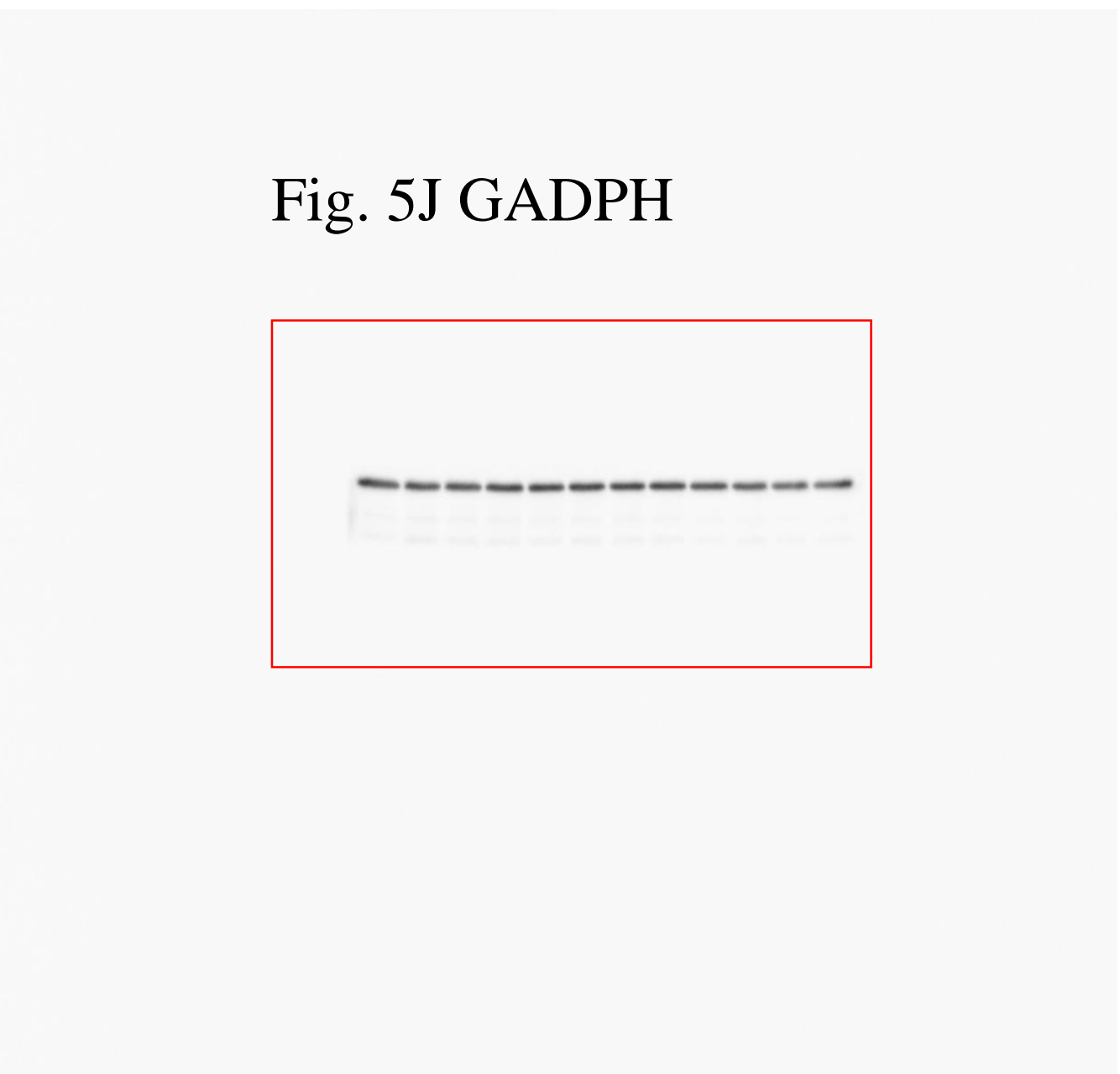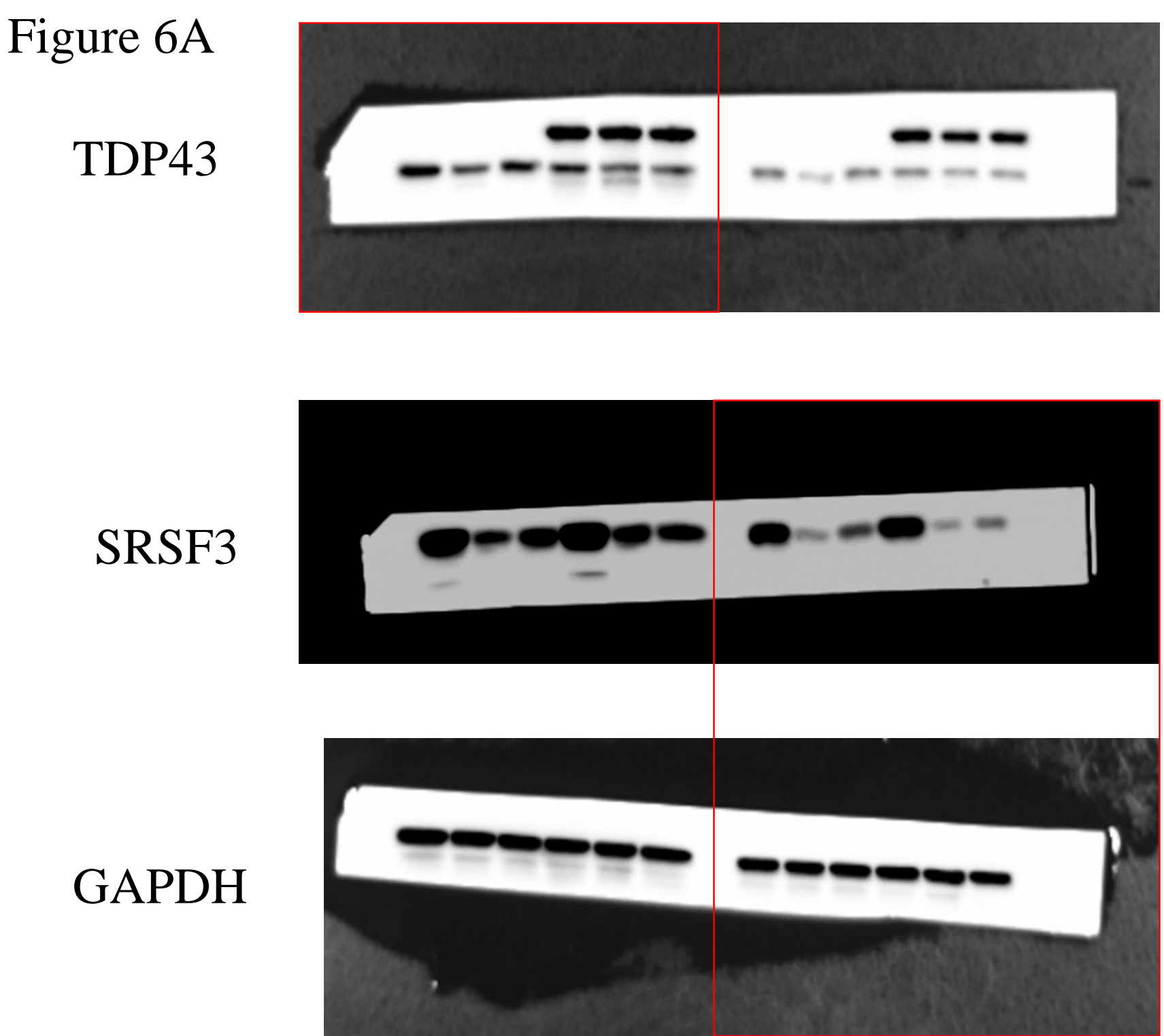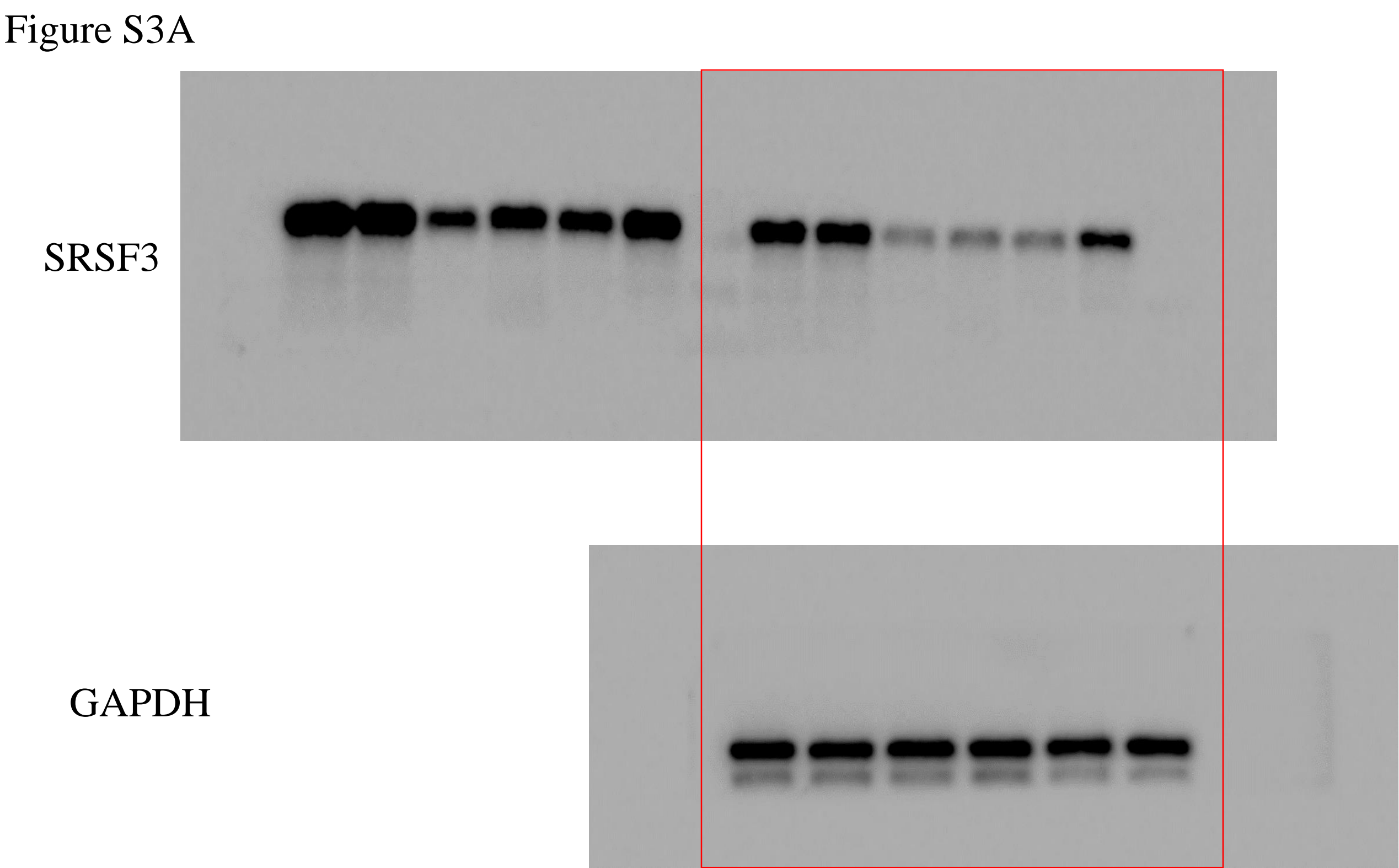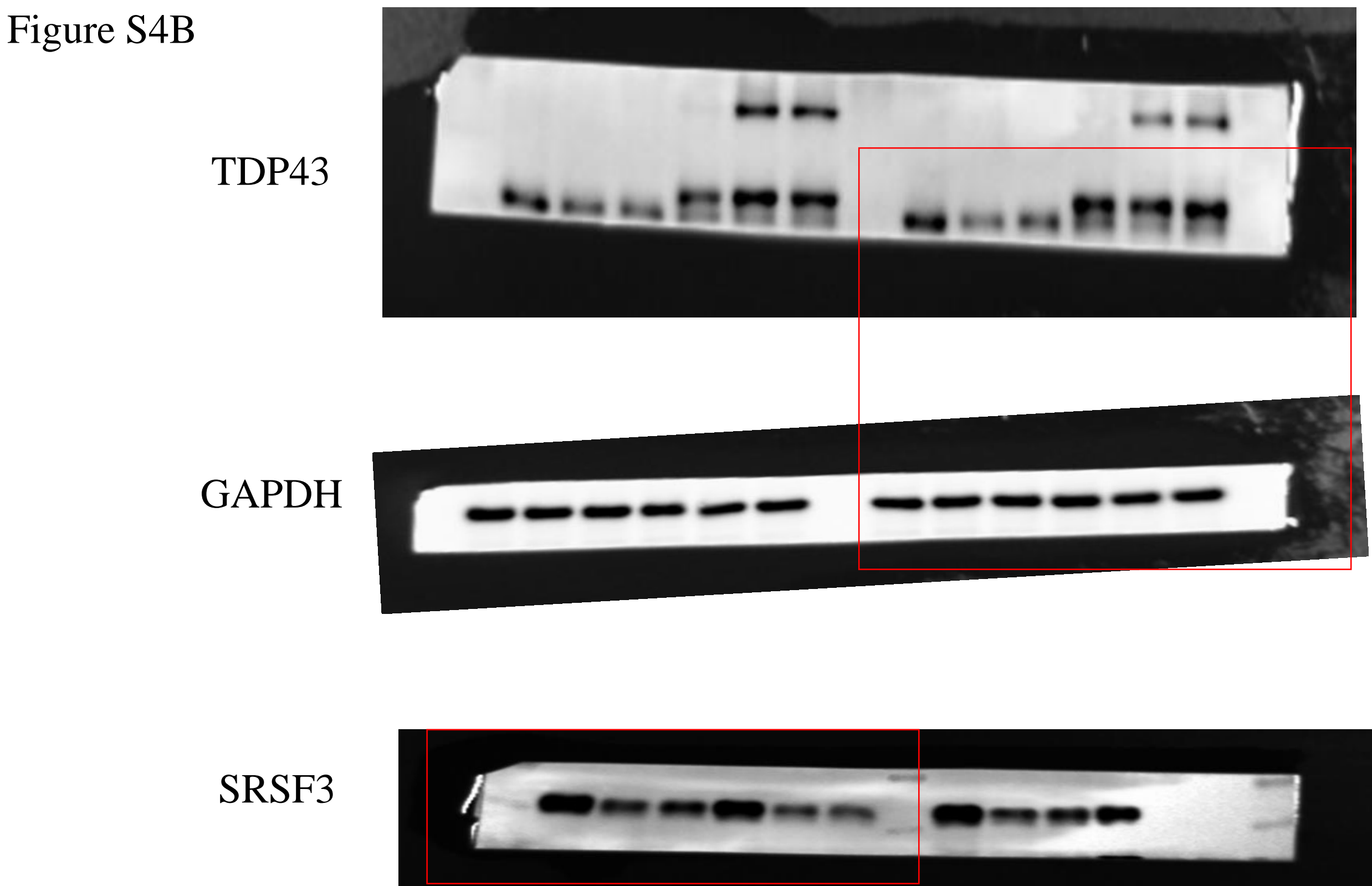

Supplement: Supplementary file 6 — Fig. WB [file 41419_2022_4867_MOESM6_ESM.pdf]
